# HyperIDS: a hypergraph learning and quantum-inspired transformer ensemble framework for IoT intrusion detection

**DOI:** 10.3389/fdata.2026.1901121

**Published:** 2026-09-01

**Authors:** Samayank Goel, Logeswari Govindaraj, Tamilarasi Kathirvel Murugan

**Affiliations:** School of Computer Science and Engineering, Vellore Institute of Technology, Chennai, Tamil Nadu, India

**Keywords:** CatBoost-XGBoost stacking, cyber threat detection, ensemble classification, explainable AI, hypergraph neural networks, intrusion detection system, IoT security, quantum-inspired feature selection

## Abstract

The unprecedented increase in the number of Internet of Things (IoT) devices has widened the attack surface of modern-day networks, making them vulnerable to various cyberattacks. Conventional intrusion detection mechanisms face challenges in identifying the subtle correlations between network traffic characteristics and providing consistent detection rates in varying heterogeneous environments. With this context, this research aims at introducing HyperIDS, an innovative Intrusion Detection System (IDS) which combines Hypergraph Learning, Quantum-Inspired Feature Selection and Optimization, and Transformer-based Ensemble Classifier for intelligent IoT cyberattack detection. First, Dual-Fitness Enhanced Gaussian Quantum Particle Swarm Optimization (DFE-GQPSO) approach is utilized to select the most relevant traffic characteristics while reducing feature space and computational cost. These selected traffic features are then converted to a hypergraph form, allowing higher order relations between different entities of the network to be captured. Following this step, Hypergraph Neural Networks (HGNN) is deployed to generate structural and relational representations from the hypergraph structure of the dataset. Long-term dependencies and attack patterns are subsequently extracted using a transformer encoder. The final classification process involves combining CatBoost and XGBoost using stacking ensemble method. In addition, a SHAP-based explainability module is incorporated to ensure transparency and trustworthiness of the developed system. In order to evaluate the proposed framework, HyperIDS is experimentally tested against two commonly used cyber security datasets, namely, TON_IoT and Bot-IoT. Experimental findings have shown superior effectiveness of HyperIDS in detecting cyber-attacks with 98.92, 98.81, 98.76, and 98.78% of accuracy, precision, recall, and F1 score, respectively on the TON_IoT dataset. Similarly, accuracy, precision, recall, and F1 scores of HyperIDS reach 99.14, 99.05, 99.01, and 99.03% on the Bot-IoT dataset. Comparison with conventional machine learning (ML), deep learning (DL), and hybrid intrusion detection techniques have proven HyperIDS's superiority in detecting cyberattacks on IoT infrastructure.

## 1 Introduction

Rapid development in the IoT has led to a transformation in the digital world, making it possible for different kinds of devices, sensors, actuators, and intelligent systems to communicate efficiently (Buczak and Guven, 2015). The IoT device has become a critical component in many areas, including smart homes, smart health, transportations, industrial automation, agriculture, and intelligent cities (Ashraf et al., 2022; Jayalaxmi et al., 2022). IoT devices help improve efficiencies in terms of data acquisition, processing, and communication. According to recent studies by industry analysts, there is an expectation that the number of connected IoT devices will surpass tens of billions in the coming few years (De Keersmaeker et al., 2023). This increase in connection numbers will result in massive growth in network traffic (Koroniotis et al., 2019). Even though IoT provides various benefits, it is equally vulnerable to a wide range of cybersecurity attacks (Apruzzese et al., 2023).

In contrast to conventional computer systems, IoT-based systems are made up of resource-constrained devices that possess low computational capabilities, insufficient memory, and energy constraints (Imran et al., 2022). Many IoT devices rely on simple or obsolete security measures that make them vulnerable to cyber threats due to the ease with which hackers are able to target them. Additionally, the heterogeneous structure of the IoT network along with its wide adoption and constant connectivity increases the threat surface significantly (De Keersmaeker et al., 2023). Such flaws in the system enable cyber attackers to launch various types of attacks, ranging from Distributed Denial of Service (DDoS), Denial of Service (DoS), reconnaissance attacks, data exfiltration, botnets, malware attacks, credential exploitation, and unauthorized access (Koroniotis et al., 2019; Meidan et al., 2018). The case of the Mirai botnet is one such example where a massive cyberattack was carried out by hacking IoT devices (Apruzzese et al., 2023).

In order to mitigate these risks, IDSs have emerged as an essential element of IoT networks in modern times. The function of an IDS is to constantly analyze any abnormality occurring in the network environment and within system activities (Liao et al., 2013). The conventional technique adopted for IDS involves matching attack signatures with pre-set ones, and this is very efficient for known attacks; nonetheless, it is less effective in recognizing unknown attacks, which is the core issue with conventional IDS techniques. On the other hand, the anomaly detection mechanism used in IDS aims to rectify this problem by adopting a learning approach regarding normal behavioral patterns (Kim et al., 2014; Meidan et al., 2018).

Advancements made in Artificial Intelligence (AI) and ML in recent times have greatly enhanced the ability to detect intrusions (Apruzzese et al., 2023). Models for ML-based IDS have the capability of automatically learning complex patterns within the traffic and distinguishing between attacks and normal network behavior (Hidayat et al., 2023; Islam et al., 2021). Classical ML algorithms such as Decision Tree, Random Forest, Support Vector Machines (SVMs), K-Nearest Neighbors (KNN), and Gradient Boosting techniques have shown remarkable results in various cybersecurity datasets (Alkadi et al., 2023). Despite the good results obtained by these algorithms, they often rely on hand-crafted features and can struggle to capture complex non-linear relations in big IoT traffic datasets (Hidayat et al., 2023).

Various DL methods have been extensively explored due to their ability to extract features automatically and learn hierarchical representations (Shone et al., 2018; Imran et al., 2022). Convolutional Neural Networks (CNNs) are extensively utilized to model the interaction of features across space in a network, while Recurrent Neural Networks (RNNs), Long Short-Term Memory (LSTM) networks, and Gated Recurrent Units (GRUs) are used to identify temporal correlations in sequential traffic flows. Models based on CNNs together with other types of RNNs have shown significant performance in intrusion detection in the IoT environment. However, traditional DL methods usually represent data in vectorial or matrix form, an approach that may not be able to sufficiently reflect the complicated relations among various network elements and traffic data (Al-Shurbaji et al., 2025). In order to address such weaknesses, a number of new methodologies based on graph representation have been proposed recently as promising ways to handle issues associated with the field of cybersecurity. The notion of Graph Neural Networks (GNNs) involves considering network flows as graph-like patterns, where nodes correspond to objects and edges represent their relations. Although graph modeling offers improvements in relation capturing, classical graphs can only model relations between two items. In real-world networks, attack patterns may involve complex interrelations between a large number of objects, protocols, connections, and properties at once (Bilot et al., 2023).

The application of hypergraph learning provides a very effective method of overcoming this issue (Gao et al., 2022). As opposed to regular graphs, the hypergraph model allows one to have a single hyperedge connecting more than two nodes at once, thus reflecting complicated high-order associations present in the network. HGNNs benefit from this capability by incorporating highly structured data into their models to identify discriminative patterns of attacks better. This enables hypergraph learning to provide remarkable advantages in detecting sophisticated cyber-attacks in the IoT environment (De Keersmaeker et al., 2023).

One of the major difficulties associated with intrusion detection in IoT is that of dealing with the high-dimensional nature of data in a network. Modern security datasets contain a large number of attributes, some of which might be unnecessary, irrelevant, or noisy. Unnecessary features increase computation cost, increase training time, and could also hamper the performance of the classifier. Thus, feature selection methods have become an integral part of IDS development (Wang et al., 2024). There are certain optimization methods, such as Particle Swarm Optimization (PSO), Genetic Algorithms (GA), Gray Wolf Optimization (GWO), and Ant Colony Optimization (ACO) that have proven to be effective in selecting relevant features from a dataset. But traditional optimization methods tend to suffer from pre-mature convergence and local optima problems.

Quantum-inspired optimization algorithms are among those solutions that can provide promising results for this purpose. By incorporating such quantum computing concepts as probability states and improved exploration mechanisms, the quantum-inspired approach provides better search and convergence properties. The DFE-GQPSO algorithm, for instance, is quite well-balanced regarding the trade-off between exploration and exploitation and enables selecting the most informative subset of features while remaining computationally efficient. This is a great option when it comes to big data problems associated with IoT-based intrusion detection systems.

In addition to efficient feature selection and structural representation learning, the modeling of long-term dependencies is another necessity in the field of intrusion detection (Vaswani et al., 2017). Network-based attacks usually unfold over a period of time and contain complex sequence dynamics that cannot be handled by simple recurrent neural networks. Even though LSTM and GRU have proven to be powerful tools in learning sequences, their sequential nature does not lend itself well to parallelism and might pose problems when handling large sequences (Fares et al., 2025; AboulEla and Kashef, 2025). More recently, Transformer architectures have emerged as a revolutionary technology for sequence modeling because of their self-attention mechanism, which learns the relationship of the whole sequence as a global context. This makes the use of Transformers especially relevant in the domain of cybersecurity where we deal with massive IoT traffic sequences.

Though significant advancements have been made in recent years through the utilization of DL, no classifier is able to produce the most accurate results on all types of attacks in all networks. As a result, ensemble methods have become increasingly popular due to their ability to increase robustness and improve generalizability. Two well-known examples of modern gradient boosting classifiers include CatBoost and XGBoost, both of which excel at providing good classification results in various problems involving machine learning (Abbas et al., 2022; Alkadi et al., 2023). Specifically, CatBoost is capable of dealing with the connections between categorical features and reduces the bias of predictions.

A new demand in modern cybersecurity systems is the need for explainability. The more IDS approaches become, the greater becomes the necessity to identify reasoning behind the decisions made by AI algorithms (Le et al., 2022). Black box models may undermine trust, accountability, and compliance with regulations. Techniques such as Explainable Artificial Intelligence (XAI) and SHapley Additive exPlanations (SHAP) help understand how features contribute to classification results. Discovering the most important features responsible for attack prediction helps to improve transparency and facilitate evidence-based security analysis (Rakine et al., 2024, 2025).

With respect to the issues stated above and existing research gaps, we present a novel IDS framework called HyperIDS, which utilizes quantum-inspired feature selection technique, hypergraph learning, Transformer encoder, and CatBoost-XGBoost stacking ensembling for intelligent IoT cybersecurity. In our model, the selected features are chosen using DFE-GQPSO. Optimized features are transformed into a hypergraph representation that allows the identification of high-order interactions between nodes of networks. Then, the hypergraph is analyzed with a HGNN, which learns rich structural information from its input. Further, a Transformer encoder exploits temporal dependencies and evolving behavior of cyberattacks (Gao et al., 2022; Wang et al., 2024). Finally, CatBoost and XGBoost models are stacked in an ensemble for intrusion classification, while SHAP explains classification results.

The efficiency of the presented HyperIDS model is tested on two popular datasets for benchmarking IoT cybersecurity, including TON_IoT and Bot-IoT. In particular, both of the used benchmark sets contain various types of attacks and realistic network traffic patterns, which allow conducting an exhaustive test of the intrusion detection systems' performance. Based on the results, it can be stated that the proposed HyperIDS algorithm demonstrates outstanding values for accuracy, precision, recall, and F1-scores compared to other ML, DL, and hybrid IDS systems.

The methodological innovation of HyperIDS lies in the unique integrated and unified five synergistic components in one unified framework of intrusion detection systems. By breaking with common IoT intrusion detection strategies that deal separately with feature selection, graph feature learning, temporal modeling, or ensemble classification, the unified HyperIDS framework seamlessly integrates the DFE-GQPSO features selection which is guided by quantum inspirations, Hypergraph Neural Network for modeling of higher-order topological information, Transformer for learning of temporal dependencies over a sequence, the ensemble classifier stacking CatBoost and XGBoost models, and SHAP for interpreting prediction decisions. Thus, the proposed framework is able to accomplish the tasks of simultaneously selecting meaningful features and avoiding feature redundancies, learning of complex higher-order interactions among network nodes, modeling long-range temporal attack behavior patterns, increasing prediction robustness by using ensemble methods and providing an intuitive explanation. Therefore, the developed framework can also solve some limitations that plague existing machine learning, deep learning, graph-based and hybrid IDS methods.

The structure of this paper is organized as follows. In section 2, previous studies regarding IoT intrusion detection, feature selection, hypergraph learning, and Transformer-based cybersecurity models are discussed. Section 3 describes the architecture of the presented HyperIDS framework in detail, as well as gives information about each component of the system. Section 4 is dedicated to discussing the experimental part of the work, including datasets, evaluation criteria, and implementation details. Section 5 covers analysis of the obtained results and performance comparison.

## 2 Related work

The fast growth of IoT networks has made cybersecurity much harder, making IDS absolutely crucial for keeping data safe. Unlike older networks, IoT has diversity, limited resources, and constantly changes—making intrusions tougher to catch. In recent years, researchers moved from basic ML to more complex methods like DL and hybrids. The latest advances include graph-based learning, transformer models, federated learning, and explainable AI, which all improve detection abilities. This section reviews those improvements but also notes the limits that led to the creation of the HyperIDS framework.

### 2.1 Early machine learning-based intrusion detection systems

Early work in intrusion detection involved mostly the application of classical ML methods for normal and attack traffic classification. Buczak and Guven (2015) provided one of the most extensive survey on ML based techniques for cybersecurity emphasizing that IDS performance depends largely on feature engineering, i.e., high quality datasets. Their work also emphasized that classical ML algorithms like decision trees, SVM and Bayesian classifiers can be useful but it has limitations in dealing with high dimensionality of network traffic data.

Sommer and Paxson (Sommer and Paxson, 2010) gave a critical analysis on the feasibility of using ML for intrusion detection, and pointed out the major drawbacks, namely the biased datasets, poor real-world representativeness of data, and discrepancy between training and real-world environments, concluding that a majority of ML based IDS perform better in lab tests than in production. Liao et al. (2013) offered a categorization of the IDS design, that classifies them into anomaly based, signature based, and hybrid approaches, and argued that scalability and adaptation are two primary problems in designing IDS. Kim et al. (2014) presented a hybrid approach using both anomaly and misuse based IDS, and showed the higher detection accuracy by combining two methods. However these methods are still relying on handcrafted features and hard to adapt to newly emerged attacks.

### 2.2 Transition to deep learning-based IDS

As computing power becomes larger, deep learning methods came to the fore over traditional machine learning methods due to their automatically learning hierarchical representations of features.

A Recurrent Neural Network (RNN) based IDS has been developed in Yin et al. (2017) where the system was able to model temporal dependence in network traffic data which was found to be performing better than traditional ML classifiers in the sequence modeling case. On the downside, RNN based methods suffer from the vanishing gradient problem and training them is computationally expensive. An autoencoder and an RNN based IDS was proposed by Shone et al. (2018) where the proposed deep learning approach enhanced feature extraction and dimension reduction. The hybrid method was proved to be more effective in intrusion detection by decreasing the redundancy of features.

Deep learning models have been shown to be very effective over shallow machine learning models for intrusion detection in IoT systems. Yet, there are also some challenges in using them such as they require high computation resources for running and a large amount of labeled data for their training process (Imran et al., 2022). Sajid et al. (2024) designed a hybrid machine learning and deep learning intrusion detection framework that out-performed pure DL or ML models by combining various learning methods. However, this approach increases computational complexity and causes high latency which limits it to real-time applications.

### 2.3 IoT dataset evolution and benchmarking

In terms of the availability of realistic datasets, it plays a key role in promoting the research of IDS. In 2017, Koroniotis et al. (2019) proposed Bot-IoT, which contains botnet attack types such as DDoS attack, reconnaissance, and data exfiltration to simulate the real botnet attack environment, and this dataset has become a general standard for the evaluation of IoT security models.

In 2018, Alsaedi et al. (2020) constructed TON_IoT dataset by combining various multi-layered data types including telemetry, network traffic, operating system logs, and IoT sensors data, which has a more holistic view for IoT environments compared to other prior datasets. In 2019, De Keersmaeker et al. (2023) performed a detailed literature review of existing public IoT datasets and pointed out several serious issues: imbalance of data, lack of variety, and artificial traffic's limitations, all of these will have a major impact on the generalization of IDS models trained with these datasets.

In 2018, Meidan et al. (2018) proposed N-BaIoT dataset and illustrated that the autoencoder could efficiently detect the IoT botnet attack. However, the attack type of their research focuses on the anomaly detection rather than complicated multi-device attack patterns.

### 2.4 Machine learning and ensemble-based IDS approaches

Several research efforts have focused on leveraging ensemble learning approaches that combine various classifiers and enhance the overall predictive performance. Abbas et al. (2022) have presented an ensemble-based IDS for IoT environments where different machine learning classifiers were used together to ensure more effective detection. Alkadi et al. (2023) analyzed optimizing ML-based IDS in IoT traffic classification and showed that ensemble approaches consistently out-perform single models but with high computational cost. Islam et al. (2021) have demonstrated that the choice of feature selection is key to increasing accuracy and reducing false alarm in ML-based IDS. Apruzzese et al. (2023) emphasized on the importance of machine learning in computer security and that ML-based IDS effectiveness depends strongly on feature engineering and dataset representativeness. Hidayat et al. (2023) carried out experimental evaluation of ML-based IDS and stated that ensemble approaches (e.g., Random Forest and Boosting) always achieve superior results compared to single classifier IDS in the detection of intrusion.

### 2.5 Hybrid deep learning and lightweight IDS models

Combining machine learning and deep learning based approaches Hybrid IDS models have been the subject of significant study and discussion due to the improved performance obtained. Imran et al. (2022) highlighted that the inclusion of deep learning into the traditional ML approach could result in improved detection rate, along with a reduction of false positives.

Al-Shurbaji et al. (2025) presents a survey of deep learning based IDS systems focused on IoT botnet detection and finds that the dominant approaches used in recent work can be broken down into Convolutional Neural Networks, Long-Short Term Memory networks, and hybrid architectures. Ashraf et al. (2022) performed a comparative study between machine learning based methods and deep learning based methods for the detection of IoT intrusions and concluded that hybrid models achieved better performance than single method models, both in terms of accuracy and robustness. Farooqi et al. (2024) has presented an optimized, light-weight botnet detection system suitable for resource constrained IoT devices but the accuracy of the system has been reduced in favor of speed by making certain simplifying assumptions in the model.

### 2.6 Graph-based learning for intrusion detection

Graph based learning has lately risen as a strong candidate for modeling interactions among network entities. Bilot et al. (2023) offered a thorough survey of GNNs applied to Intrusion Detection Systems and show how GNNs can model interactions between hosts, packets and flows.

Nonetheless, standard graph based approaches restrict interactions to only pairwise entities and do not consider interactions between multiple entities that are omnipresent in IoT networks. This motivates the use of hypergraph based learning methods.

### 2.7 Hypergraph neural networks and higher-order learning

Hypergraph neural networks generalize graph based methods, allowing to model the interaction of more than two nodes at once. Gao et al. (2022) present HGNN+ that improves representation learning through modeling complicated dependencies in non-Euclidean data representations. The interactions in IoT networks are of the form that cannot be properly represented in conventional graphs, because several nodes interact at the same time. Learning using hypergraphs represents IoT traffic and networks in a more expressive way. On the other hand, hypergraph learning involves huge computational cost and efficiency has to be attained using a feature selection and dimensionality reduction approach.

### 2.8 Transformer-based intrusion detection systems

Self-attention mechanisms have helped Transformer architectures transform the way sequences are modeled. Vaswani et al. (2017) originally presented the Transformer model which lacks recurrence and can process sequence data in parallel. Fares et al. (2025) implemented a Swin Transformer-LSTM model to apply to IoT intrusion detection, resulting in enhanced features extraction and classification capability. AboulEla and Kashef (2025) also found that transformer based IDS can outperform conventional deep learning models for network traffic classification. Although Transformer models yield accuracy higher than average, the complexity and resource usage of transformer models are very high and selection methods must be used carefully to make transformer models feasible in the IoT environment.

### 2.9 Optimization techniques in IDS

Optimization techniques are also beneficial to increase IDS performance by helping in feature selection and tuning of parameters. Wang et al. (2024) used quantum-inspired swarm optimization algorithms and successfully applied them on various complex problems like cybersecurity. The quantum-inspired technique uses its strength to improve exploration and prevent pre-mature convergence than usual swarm intelligence algorithms. This technique is quite effective for high-dimensional data such as in IoT.

### 2.10 Explainable AI in intrusion detection systems

Explainability is one of the core requirements for modern IDSs in order to provide trust and transparency. Le et al. (2022) proposed a SHAP-based explanation framework for ensemble tree models in intrusion detection systems in order to provide feature-level interpretation.

Methods for interpretable AI, such as SHAP can be used to increase model transparency by providing a measure for the contribution of individual features to a final prediction.

### 2.11 Federated learning for privacy-preserving IDS

In order to overcome the security concerns of intrusion detection, federated learning is developing rapidly. The paper given by Alsaleh et al. (2024) discussed comprehensive study on federated learning-based IDS for heterogeneous IoT networks by investigating some challenges, such as communication burden and non-IID data distribution. Alsaleh et al. (2025) employed federated learning with BiLSTM in lightweight IDS that aware of heterogeneous information, which can further decrease the communication and resource load. A hybrid federated learning approach that can increase the privacy and scalability of IDS in IoT is proposed by Agili et al. (2025). A detailed study on systematic literature review on federated learning-based IDS, especially for the key challenges of model convergence, data heterogeneity and limited resource, is illustrated by Kale et al. (2026).

Although the recent graph and transformer-based models have yielded promising results on this task, most of the current studies concentrate only on parts of the intrusion detection pipeline. GNN based models are effective to capture the relationships between pairs of entities, but cannot represent higher-order relations in complicated IoT attack patterns effectively. HGNN based methods tackle this issue by modeling the multi-way relations, while mostly concerned about structural representation learning and lack of modeling the time progression of attacks. On the other hand, transformer based models are powerful for modeling long-range temporal dependencies by using attention, but often assume vectorized feature representation without exploiting the structural dependencies in interconnected samples. Many HGNN-transformer methods lack the advanced features selection methods and single classifiers, while poor on the interpretability of the models. In this work, we proposed a novel HGNN-transformer model, named HyperIDS, which jointly leverages quantum-inspired feature optimization, hypergraph learning on high-order relations, attention modeling of temporal dependencies, and CatBoost-XGBoost stacking classification with SHAP explainability to address the above shortcomings of the current studies. A comparison of key IDS approaches for IoT is presented in Table 1.

**Table 1:** Comparison of key IDS approaches for IoT.

Approach	Key techniques	Strengths	Limitations
Traditional ML IDS (Buczak and Guven, 2015; Sommer and Paxson, 2010; Liao et al., 2013; Kim et al., 2014)	SVM, Decision Trees, Bayesian models, anomaly + misuse detection	Simple, fast training, interpretable	Poor generalization, high feature dependency, weak for high-dimensional IoT data
Classical deep learning IDS (Shone et al., 2018; Yin et al., 2017; Sajid et al., 2024; Imran et al., 2022)	RNN, CNN, Autoencoder, deep neural networks	Automatic feature learning, high accuracy	High computational cost, requires large datasets, poor explainability
IoT dataset-based IDS (Koroniotis et al., 2019; Alsaedi et al., 2020; De Keersmaeker et al., 2023; Meidan et al., 2018)	Bot-IoT, TON_IoT, N-BaIoT, multi-layer telemetry analysis	Realistic attack scenarios, benchmark standardization	Dataset imbalance, synthetic bias, pre-processing complexity
Machine learning and ensemble IDS (Apruzzese et al., 2023; Hidayat et al., 2023; Abbas et al., 2022; Alkadi et al., 2023)	Random forest, ensemble stacking, boosting models	High accuracy, robust classification, handles non-linearity	Limited temporal modeling, interpretability issues, higher complexity
Hybrid ML–DL IDS (Sajid et al., 2024; Imran et al., 2022; Al-Shurbaji et al., 2025; Ashraf et al., 2022; Farooqi et al., 2024)	CNN + LSTM, ML + DL fusion, lightweight DL models	Improved accuracy, reduced false positives, adaptable	High training cost, latency issues, limited real-time deployment
Graph-based IDS (Bilot et al., 2023)	Graph neural networks (GNNs)	Captures relational dependencies, improves anomaly detection	Limited to pairwise relationships, scalability issues
Hypergraph learning IDS (Gao et al., 2022)	Hypergraph neural networks (HGNN+)	Models higher-order relationships, better structural representation	High computational cost, requires optimized feature selection
Transformer-based IDS (Vaswani et al., 2017; Fares et al., 2025; AboulEla and Kashef, 2025)	Self-attention, swin transformer, transformer encoders	Captures long-range dependencies, high accuracy	Computationally expensive, requires optimization for IoT deployment
Optimization-based IDS (Wang et al., 2024)	Quantum-inspired swarm optimization (QPSO variants)	Better feature selection, avoids local minima	Complexity in tuning, computational overhead
Explainable IDS (XAI) (Le et al., 2022)	SHAP-based feature attribution	Improves transparency, interpretable predictions	Additional computation, *post-hoc* explainability limitations
Federated learning IDS (Alsaleh et al., 2024, 2025; Agili et al., 2025; Kale et al., 2026)	FL, BiLSTM, decentralized training, privacy-preserving learning	Preserves privacy, scalable, distributed learning	Communication overhead, non-IID data, convergence issues

### 2.12 Research gap analysis

Based on the thorough literature survey we found the following gaps:

- The combination of feature optimization with deep representation learning has not been explored significantly.
- The hypergraph-based higher order relation modeling for IDS have not been utilized much.
- High complexity for transformer-based IDS models has been a problem.
- Combination of ensemble learning with structural representation learning has not been explored enough.
- The recent sophisticated hybrid IDS models are lacking explainability.
- Federated learning only concerned about the privacy rather than detection rate.
- Existing models are difficult to scale in the heterogeneous IoT environments.

The following components are employed by the proposed HyperIDS to overcome the above shortcomings:

- DFE-GQPSO for feature selection.
- Hypergraph Neural Networks for high-order relationships.
- Transformer encoder to capture long range dependency.
- Stacking Ensemble (CatBoost, XGBoost) for better classification performance.
- Explainability module based on SHAP.

## 3 Proposed HyperIDS framework

### 3.1 System overview

**Figure 1:**
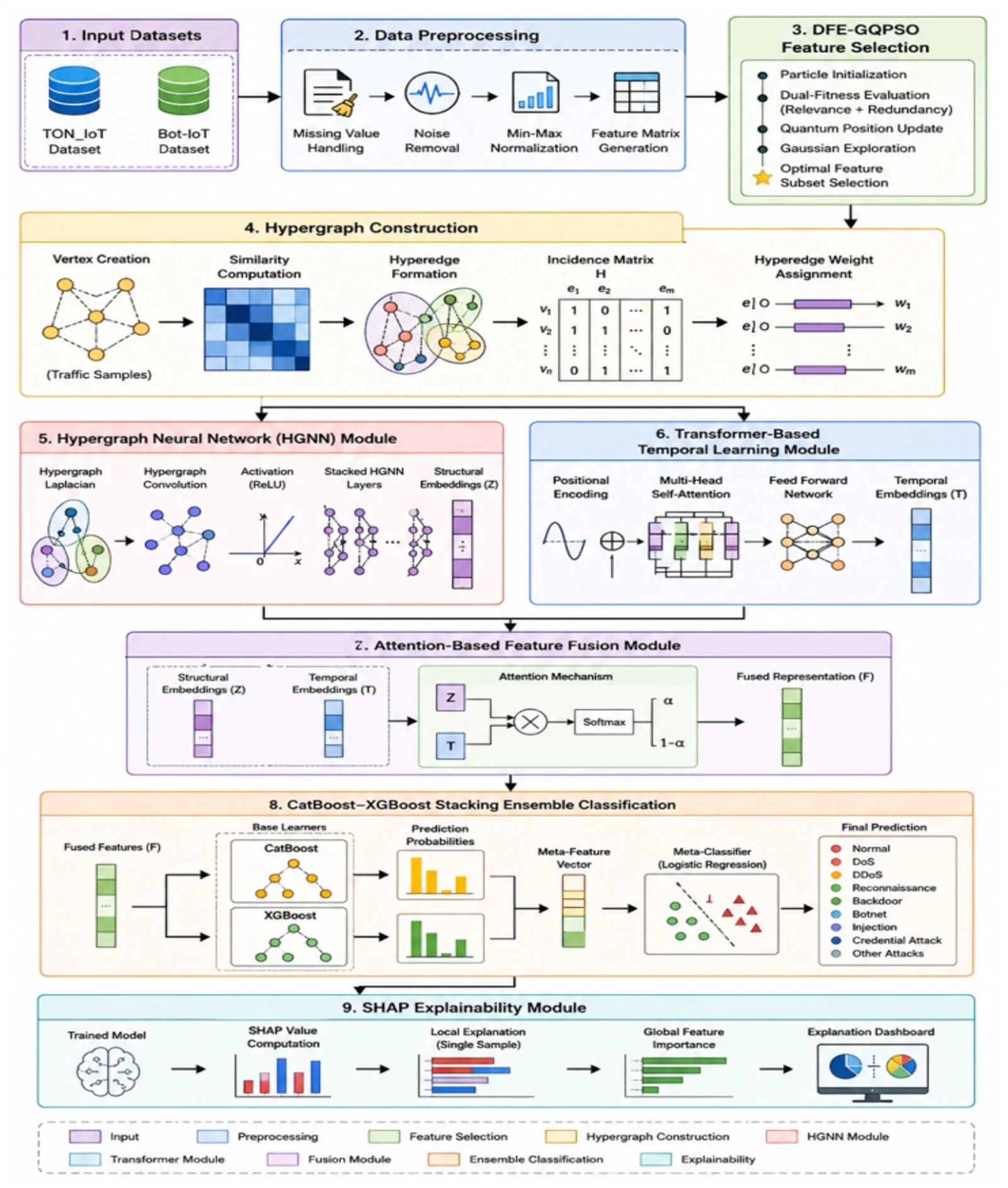
Proposed HyperIDS architecture forintelligent iot intrusion detection.

The growing sophisticated nature of attacks on IoT settings demands that there is an urgent need to build intelligent intrusion detection systems which can detect malicious events with good accuracy while remaining computationally efficient. Typical Machine learning and Deep learning techniques process network traffic as individual data instances and can be unaware of the intricate higher-order relations which inherently exists between entities, communication session, and protocol relationships. High-dimensional traffic feature representation and changing threat vectors have made it very difficult to detect attacks. In this paper, we propose HyperIDS, a novel framework that integrates DFE-GQPSO, HGNNs, Transformer-based temporal modeling, CatBoost-XGBoost stacking ensemble classification and SHAP-based explainability. As showed in Figure 1, the proposed HyperIDS is designed to accurately detect malicious IoT traffic by incorporating quantum-inspired feature selection, hypergraph representation learning, Transformer-based temporal modeling, and ensemble classification.

In essence, HyperIDS is built upon the following six modules: (1) Pre-processing module will remove noisy and normalize feature distributions for raw network traffic from TON_IoT and Bot-IoT datasets. (2) DFE-GQPSO is proposed to discover the subset of significant features while shrinking dimensions. (3) Reduced features will be represented into a hypergraph form to model the higher-order relationships between traffic flow. (4) HGNN is implemented to learn the structural attack representations from constructed hypergraph. (5) Transformer encoders can capture the temporal relationship between flows and produce feature embedding in context. (6) The final intrusion classification is achieved by a CatBoost-XGBoost stacking ensemble and the interpretability of classification results is provided by the SHAP module.

The core methodological contribution of HyperIDS is its efficient incorporation of optimization, higher-order representation learning, modeling temporal dependencies, ensemble classification, and interpretable artificial intelligence into an integrated, end-to-end framework. In contrast to separate handling of these elements, the present framework employs a mutually complementary learning process wherein each of these units improves the performance of later ones. Before constructing hypergraphs, features redundant for detection are removed using DFE-GQPSO, the complex higher-order structural relationships of traffic samples are seized by HGNN, remote time dependencies are modeled by the Transformer encoder, the accuracy of the classifier is augmented by the stacking ensemble of CatBoost-XGBoost, and the SHAP module interprets the decision rationale behind predictions, thereby significantly boosting the efficiency, accuracy and transparency of IoT intrusion detection.

Let D represent the IoT traffic dataset:


$$\begin{array}{l} D={\left\{\left(x_{i},y_{i}\right)\right\}}_{i=1}^{N} \end{array}$$ (1)

In Equation 1 $x_{i}\in {\mathbb{R}}^{m}\;$denotes the feature vector for the ith traffic sample and *y*_*i*_denotes the class label of the ith traffic sample. The parameter *m* denotes the number of traffic features and N indicates the number of traffic samples. Equation 1 presents the full dataset in the HyperIDS framework. Each feature vector encodes details about the communication behaviors, packet characteristics, protocol features, and network flow features. The aim of the designed framework can be framed as follows:


$$\begin{array}{l} Ŷ=F\left(D\right) \end{array}$$ (2)

where F(.) represents the HyperIDS learning framework and Ŷ denotes the output of attack classification labels. Equation 2 indicates that HyperIDS performs the non-linear mapping between the network traffic observation and attack classes via feature optimization, structural representation learning, temporal modeling, and ensemble classification.

### 3.2 Data pre-processing module

TON_IoT and Bot-IoT datasets present heterogeneous traffic features stemming from several IoT devices and network services. Because features have different scales and statistical distributions, a data pre-processing step is required before features can be optimized and a classifier can be trained. When features are not scaled properly, they can influence optimization algorithms adversely.

#### 3.2.1 Data cleaning

The raw traffic records may contain missing values, redundant entries, and noisy entries. The value missing entries were imputed using mean-value imputation as in Equation 3:


$$\begin{array}{l} x_{miss}=\frac{1}{n}\overset{n}{\underset{i=1}{\sum }}x_{i} \end{array}$$ (3)

where *x*_*miss*_ indicates the imputed value, *x*_*i*_ represents the valid values corresponding to the feature, and n is the number of available entries. Equation 3 prevent the information missing from influencing learning process in following stages.

#### 3.2.2 Min-max normalization

To make each feature having the same scales, we perform min-max normalization.


$$\begin{array}{l} x_{norm}=\frac{x-x_{\min}}{x_{\max}-x_{\min}} \end{array}$$ (4)

where *x* is the original value of the attribute, *x*_min_ is the min value, and *x*_max_ is the max value. As can be seen from Equation 4, all attributes are scaled into the range [0, 1], and therefore the values of different attributes have similar effects in both optimization and classification stage.

#### 3.2.3 Feature matrix formation

After processing, the normalized traffic matrix can be expressed as:


$$\begin{array}{l} X=\left[\begin{array}{cccc} x_{11} & x_{12} & \cdots & x_{1m}\\ x_{21} & x_{22} & \cdots & x_{2m}\\ \vdots & \vdots & \ddots & \vdots \\ x_{N1} & x_{N2} & \cdots & x_{Nm} \end{array}\right] \end{array}$$ (5)

where, *X* represents the normalized traffic matrix, N stands for the total number of samples, and m represents the number of features. The matrix defined in Equation 5 is fed to the DFE-GQPSO feature selection module.

### 3.3 DFE-GQPSO-based feature selection

The overall performance of IDS is significantly related to input features. Since high-dimensional network traffic datasets usually contain irrelevant and redundant attributes which will greatly increase the calculation time and decrease classification performance. So, HyperIDS used the algorithm DFE-GQPSO. In order to resolve this problem, the optimizer we propose in this paper has applied the quantum evolution on particle along with Gaussian perturbation scheme. The DFE-GQPSO updates the state using probabilities and adaptive exploration rather than just using the velocity for updating.

#### 3.3.1 Particle representation

Each particle denotes a subset of possible features to choose:


$$\begin{array}{l} P_{i}=\left[p_{i1},p_{i2},\ldots ,p_{im}\right] \end{array}$$ (6)

*P*_*i*_ denotes the *i*^*th*^ particle and *p*_*ij*_ ϵ {0,1} is the state of the j^*t*^*h* feature in Equations 6, 1 represents selecting the *j*^*t*^*h* feature, otherwise 0 indicates not selecting it.

#### 3.3.2 Dual-fitness objective function

We are minimizing the number of selected features and the classification accuracy at the same time:


$$\begin{array}{l} Fitness=\alpha Acc+\left(1-\alpha \right)\left(1-\frac{|S|}{m}\right) \end{array}$$ (7)

Where *Acc* refers to classification accuracy, ∣*S*∣ stands for number of selected features, and *m* refers to total features, and it influences the detection performance vs dimensional reduction trade-off. From (7), we can see that the search aims to finding a compressed set of features to achieve high predictive power.

The calculation of classification accuracy is given by Equation 8:


$$\begin{array}{l} Acc=\frac{TP+TN}{TP+TN+FP+FN} \end{array}$$ (8)

where *TP* denotes true positives, *TN* denotes true negatives, *FP* denotes false positives, and *FN* denotes false negatives.

#### 3.3.3 Mean best position

Quantum mean best position is calculated as:


$$\begin{array}{l} mbest=\frac{1}{M}\overset{M}{\underset{i=1}{\sum }}P_{i}^{best} \end{array}$$ (9)

Here *M* is size of the swarm and $P_{i}^{best}$ is the best personal position. It can be observed from Equation 9 that mean best position represents information of the entire swarm.

#### 3.3.4 Quantum position update

Particle positions can be updated as,


$$\begin{array}{l} P_{i}^{t+1}=mbest\pm \beta |P_{i}^{t}-Gbest|ln\left(\frac{1}{u}\right) \end{array}$$ (10)

where $P_{i}^{t}$ is the position of particle at time t, *Gbest* is best position of the swarm, β is the contraction-expansion coefficient, and *u* is a uniform random variable. From Equation 10, it can be seen that regions of promising search can be explored along with maintaining stability of convergence.

#### 3.3.5 Gaussian exploration

To avoid pre-maturely converging, a Gaussian perturbation mechanism is introduced:


$$\begin{array}{l} P_{i}^{new}=P_{i}+N\left(0,\sigma^{2}\right) \end{array}$$ (11)

In this equation, $N\left(0,\sigma^{2}\right)$ is a Gaussian noise term, and is standard deviation. Equation 11 can effectively improve the search space diversity and local optimization performance.

### 3.4 Hypergraph construction module

Classical graph-based IDS model pairs of relations between traffic elements. In fact, cyber-attacks do involve multi-devices, flows, protocols, and patterns at the same time. So, in order to seize those higher-order correlations, HyperIDS transforms pre-optimized traffic information to a hypergraph representation. Hypergraph definition is: Equation 12


$$\begin{array}{l} H=\left(V,E,W\right) \end{array}$$ (12)

where V is the set of vertices, E is the set of hyperedges, and W represents hyperedge weights. Unlike traditional graphs, where an edge is defined between two vertices, hyperedges connect a variable number of vertices at once.

In the proposed HyperIDS framework, the traffic samples optimized by the DFE-GQPSO feature selection module are then transformed to a hypergraph format. Each traffic sample is represented by a vertex. Multiple traffic samples with similar communication characteristics in the optimized feature space are connected by hyperedges. For instance, similarity between traffic samples is calculated using the Euclidean distance between the respective feature vectors obtained in the DFE-GQPSO optimization. A particular traffic sample is then connected to its k-nearest neighbors to create a single hyperedge consisting of the particular traffic instance and its neighborhood instances. In this manner, a hyperedge connects more than two vertices at a time to represent higher-order correlations between network flows. Finally, weights based on similarities are assigned to each created hyperedge by the Gaussian kernel to provide high weights to more correlated traffic samples than to poorly correlated samples, hence the formation of a weighted hypergraph to keep structural relations between the IoT traffic instances as input to the HGNN-based representation learning module.

#### 3.4.1 Hypergraph incidence matrix

The incidence relationship is defined as follows:


$$H(v,e)=\{\begin{array}{cc} 1, & v\in e\\ 0, & otherwise \end{array}$$ (13)

where *v* and *e* are a vertex and a hyperedge respectively. Equation 13 finds which vertex belong to which hyperedge.

#### 3.4.2 Vertex degree

The degree of every vertex is calculated as:


$$\begin{array}{l} d\left(v\right)=\underset{e\in E}{\sum }w\left(e\right)H\left(v,e\right) \end{array}$$ (14)

where *w*(*e*) is a hyperedge weight. Equation 14 determines how strong a vertex is connected to hyperedges.

#### 3.4.3 Hyperedge degree

The hyperedge cardinality is calculated as:


$$\begin{array}{l} \delta \left(e\right)=\underset{v\in V}{\sum }H\left(v,e\right) \end{array}$$ (15)

where δ(*e*) is the hyperedge degree. Equation 15 measures how many vertices are connected to hyperedge.

#### 3.4.4 Hyperedge weight assignment

The hyperedge weight based on the similarity is:


$$\begin{array}{l} w\left(e\right)=exp\left(-\frac{∥x_{i}-x_{j}{∥}^{2}}{\sigma^{2}}\right) \end{array}$$ (16)

where *x*_*i*_ and *x*_*j*_ are the traffic instances and σ controls neighborhood sensitivity. Equation 16 shows that highly similar traffic patterns get greater weights.

The hypergraph construction process has four consecutive steps. First, the optimized feature vectors obtained from the DFE-GQPSO feature selection module are taken as hypergraph vertices, and each vertex is one traffic sample characterized with the chosen discriminative features. Second, the relationship between neighborhood is built using k-nearest neighbors (k-NN) rule in the optimized feature space to select traffic samples with the most similar structures. Third, hyperedges are constructed to connect each reference traffic sample with its neighboring samples, by which a hyperedge is allowed to contain several similar traffic samples at the same time and can learn higher-order interactions that cannot be described by pair graphs. Finally, the Gaussian similarity weighting to every hyperedge is assigned as Equation 16 such that traffic group with highly similar is granted greater weight. The weighted hypergraph can better retain the complex structural dependencies of the IoT traffic flows, and is passed into the next module of the HGNN for the higher-order representation learning.

Dynamic Hypergraph Update for Streaming IoT Traffic: The hypergraph we use in this paper is built on top of offline benchmark datasets for evaluation, however, our HyperIDS can be readily extended for dynamic environments where traffic flows in a continuous manner. Instead of rebuilding the entire hypergraph when new traffic samples arrives, we insert each arriving traffic instance as a new vertex in the hypergraph, and the memberships of the newly formed hyperedges are determined with regard to a small number of nearest traffic samples detected based on the k-NN similarity measures utilized during hypergraph construction. Thus, only those local row regions of the hypergraph incidence matrix, and the vertex/hyperedge degree matrices and the local Laplacian values corresponding to those rows, will need to be updated while the overall hypergraph structure is preserved. The cost reduction incurred from such localized update greatly accelerates the process of dealing with the continuously arriving traffic data. Alternatively, incoming traffic samples may accumulate in a sliding window or a mini-batch before their local updates on the hypergraph occur, thus trading between efficiency and quality for real-time detections. Such a new weighted hypergraph can be directly plugged in our HGNN block, instead of being reconstructed on-the-fly from scratch.

### 3.5 Hypergraph neural network feature learning

Although the hypergraph constructed in the above phase does represent higher-order relation among traffic events, just constructing the hypergraph itself can't ful-fill the goal of attack detection. It's necessary to transfer the sophisticated structure information contained in hypergraph to discriminative features, which requires transforming hypergraph into a feature space where we can learn better features. Hence in the proposed HyperIDS framework, HGNN is employed to model topological dependence and higher-order attack structure in hypergraph.

Differ from GNN that only considers pairwise relations, HGNN propagates information over the hyperedges that contain multiple vertices at the same time. This capability enables HGNN to capture concurrent attack actions, distributed attack actions, and intricate communication relations in many IoT botnets and state-of-art cyber-attacks. We define the constructed hypergraph as in Equation 17:


$$\begin{array}{l} H=\left(V,E,W\right) \end{array}$$ (17)

Here, V stands for the vertex set, E for the hyperedge set, and W stands for the hyperedge weights. In Section 3.4, we define each vertex to be one traffic sample, and hyperedges for multiple interrelated traffic instances.

#### 3.5.1 Hypergraph degree matrices

The vertex degree matrix is given by:


$$\begin{array}{l} D_{v}\left(i,i\right)=\underset{e\in E}{\sum }w\left(e\right)H\left(i,e\right) \end{array}$$ (18)

where the diagonal term of *D*_*v*_ represents the degree for hyperedge i and weighted hyperedge degrees are considered (if *w*(*e*) is present). The incidence relation *H*(*i, e*) is a binary value and is defined as: *H*(*i, e*) = 1 if hyperedge i is incident to vertex e and 0 otherwise. Equation 18 calculates the contribution of hyperedges incident to each vertex i. The hyperedge degree matrix *D*_*e*_(*e, e*) is computed as:


$$\begin{array}{l} D_{e}\left(e,e\right)=\underset{v\in V}{\sum }H\left(v,e\right) \end{array}$$ (19)

where *D*_*e*_ represents hyperedge degree matrix. From the Equation 19, we could compute how many vertices are connected together at each hyperedge.

#### 3.5.2 Hypergraph laplacian construction

In order to realize information propagation over high-order structures, normalized hypergraph Laplacian is constructed:


$$\begin{array}{l} L=D_{v}^{-1/2}HWD_{e}^{-1}H^{T}D_{v}^{-1/2} \end{array}$$ (20)

Here *L* represents normalized hypergraph Laplacian, *H* the incidence matrix, *W* the hyperedge weight matrix, *D*_*v*_ the vertex degree matrix and *D*_*e*_ the hyperedge degree matrix. As presented in Equation 20, this Laplacian can seize the structural relationships between the traffic samples and collect information through hypergraph in an effective way.

#### 3.5.3 Hypergraph convolution

HGNN propagates information through stacked convolution layers. The hidden representation of layer l+1 can be formulated as:


$$\begin{array}{l} X^{\left(l+1\right)}=\sigma \left(LX^{\left(l\right)}W^{\left(l\right)}\right) \end{array}$$ (21)

where *X*^(*l*)^ is the feature at layer *l*, *W*^(*l*)^ is the learnable parameter, and σ(.) is ReLU function. As seen in Equation 21, the convolution operation aggregated neighbors information through hyperedge while maintaining structural relationship between traffic samples. And ReLU function can be represented as:


$$\begin{array}{l} \sigma \left(x\right)=max\left(0,x\right) \end{array}$$ (22)

where *x* denotes the input activation value. Equation (22) introduces nonlinearity into the HGNN model and improves representation learning capability.

#### 3.5.4 Hypergraph embedding generation

After repeated convolution operations, we obtained the final embedding matrix:


$$\begin{array}{l} Z=HGNN\left(X,H\right) \end{array}$$ (23)

Where Z is the hypergraph embedding matrix learned. Equation 23 has resulted in abstract structural features which can differentiate benign traffic from attacks. They preserve: Topological relationship, Higher-order interaction, Communication dependency, Attack propagation pattern and hence a well-represented profile of the IoT traffic.

When deployed online, HGNN does not need to be retrained from scratch when hypergraphs are updated incrementally. Because message propagation mainly depends on hyperedge neighborhoods locally, only the embeddings of new added vertices and the vertices connected to these new vertices are updated, while those for rest areas remain unchanged. The localized embedding updates can significantly speed up inference and allow HyperIDS to scale well to the very large-scale dynamically evolving IoT networks.

### 3.6 Transformer-based temporal learning

Even though HGNN is able to model the relationship between traffic samples according to their structure, it is unable to learn about their temporal relation. In fact, cyber-attacks have time-dependent characteristics. Attacks occur sequentially in various stages: exploration, intrusion, privilege escalation and information leakage, therefore time modeling is crucial to effectively detect intrusions. Since traditional recurrent neural network is difficult to learn long-term dependencies and it has limitation to parallel learning, a Transformer encoder, which applies self-attention mechanism to capture time dependency information on a global scale, is used in HyperIDS.

**Sequence generation from HGNN embeddings:** after learning the higher-order relations in the generated hypergraph, the HGNN module outputs a structural embedding for each sample of network traffic. The input to the Transformer must be in the form of sequences, so the above individual embeddings of samples are ordered chronologically, according to network traffic flow. In particular, HGNN embeddings are divided into fixed-length sequences via a sliding window mechanism, each window includes the recent network traffic samples occurred within some time duration. The window size is defined by L, and for each window, its HGNN embeddings in temporal order are concatenated into a sequence which is given as input to the Transformer encoder. As new network traffic samples arrive, the sliding window shifts forward by one or more steps, creating the overlapping sequences that retain the temporal continuity and efficient learning the evolving attack behavior, so that the Transformer can capture short and long term correlations of subsequent events in the network traffic.

The input sequence is the sequence of embeddings from HGNN:


$$\begin{array}{l} Z=\left[z_{1},z_{2},\ldots ,z_{n}\right] \end{array}$$ (24)

where *z*_*i*_ denotes the sequence of embeddings corresponding to the *i*^*th*^ traffic sample, and Equation 24 is the input sequence for the Transformer module.

#### 3.6.1 Positional Encoding

As Transformers have no sequence order built into the model. Positional encodings are used. The definition of the sinusoidal positional encoding is given by:


$$\begin{array}{lll} PE\left(pos,\;2i\right) & = & \sin\left(\frac{pos}{10000^{2i/d}}\right) \end{array}$$ (25)

$$\begin{array}{lll} PE\left(pos,\;2i+1\right) & = & \cos\left(\frac{pos}{10000^{2i/d}}\right) \end{array}$$ (26)

Where *pos* is the sequence position and *i* is the dimension of the embedding while *d* is the embedding size. Equations 25, 26 allow for positional information to be conveyed in a manner which also retains order information between samples of traffic.

The final input to the Transformer becomes:


$$\begin{array}{l} T_{0}=Z+PE \end{array}$$ (27)

In Equation 27, *T*_0_ represents the initial Transformer representation.

#### 3.6.2 Self-attention mechanism

For a Transformer, the computations for the Query, Key and Value matrices is done as shown in Equations 28–30


$$\begin{array}{lll} Q & = & T_{0}W_{Q} \end{array}$$ (28)

$$\begin{array}{lll} K & = & T_{0}W_{K} \end{array}$$ (29)

$$\begin{array}{lll} V & = & T_{0}W_{V} \end{array}$$ (30)

Here, *W*_*Q*_, *W*_*K*_, and *W*_*V*_ represent the matrices containing learnable parameters. The attention score is calculated as:


$$\begin{array}{l} Attention\left(Q,K,V\right)=Softmax\left(\frac{QK^{T}}{\sqrt{d_{k}}}\right)V \end{array}$$ (31)

Here, *d*_*k*_represents the dimension of key. In the Equation 31, the self-attention mechanism is to allow each traffic sample to attend to all the traffic samples in the sequence, thereby providing a long-range interaction to attack dependencies that are normally captured poorly by conventional recurrent networks.

#### 3.6.3 Multi-head attention

To extract different features of attack. We utilize multiple attention heads:


$$\begin{array}{l} head_{i}=Attention\left(Q_{i},K_{i},V_{i}\right) \end{array}$$ (32)

In Equation 32 *head*_*i*_ denotes output of *i*^*th*^ attention head.

We concatenate these output:


$$\begin{array}{l} MHA=Concat\left(head_{1},\;head_{2},\ldots ,\;head_{h}\right)W_{O} \end{array}$$ (33)

where *h* is the number of attention heads, and *W*_*O*_ is the output projection matrix. Equation 33 permits learning multiple patterns of attack and temporal dependencies at the same time.

#### 3.6.4 Feed forward network

The output of Transformer is further refined by:


$$\begin{array}{l} FFN\left(x\right)=W_{2}\left(ReLU\left(W_{1}x+b_{1}\right)\right)+b_{2} \end{array}$$ (34)

In the Equation 34, *W*_1_ and *W*_2_ are trainable parameters and *b*_1_, *b*_2_ are trainable biases. By Equation 34, it increases representation ability and provides another non-linear operation.

The final temporal representation is represented as in Equation 35:


$$\begin{array}{l} T=Transformer\left(Z\right) \end{array}$$ (35)

where *T* denotes the temporal embedding matrix.

### 3.7 Attention-based feature fusion

The HGNN embeddings represent the structural information and the Transformer embeddings represent the temporal information. None of the two individual representations can describe the attack behavior. Consequently, HyperIDS uses an attention-based fusion mechanism to combine structural and temporal knowledge.

Let:


$$\begin{array}{lll} F_{s} & = & Z \end{array}$$ (36)

$$\begin{array}{lll} F_{t} & = & T \end{array}$$ (37)

In Equations 36 and 37, *F*_*s*_ is the structural embeddings and *F*_*t*_ represents the temporal embeddings.

The fusion weight is calculated as:


$$\begin{array}{l} \alpha =\frac{\exp\left(F_{s}\right)}{\exp\left(F_{s}\right)+\exp\left(F_{t}\right)} \end{array}$$ (38)

In Equation 38, α denotes attention weight.

The final fused representation becomes:


$$\begin{array}{l} F=\alpha F_{s}+\left(1-\alpha \right)F_{t} \end{array}$$ (39)

where *F* denotes the integrated feature representation. As indicated by Equation 39, the fusion mechanism dynamically balances structural and temporal information based on their relative importance. The resulting feature representation is subsequently forwarded to the CatBoost-XGBoost stacking ensemble for attack classification.

### 3.8 CatBoost–XGBoost stacking ensemble classification

Once these features are extracted with the structure and temporal information from HGNN and Transformer modules, it is essential to perform binary classification (normal vs. Attack) to the extracted combined feature vector. In this context, machine learning and deep learning based classifiers may individually produce reasonable accuracy, however, none of the individual classifiers perform best for all attack classes. The nature and distributions and feature dependency relationships differ from one attack class to another. Therefore, single classifier may not be robust for all IoT attack types. In order to tackle this limitation, the HyperIDS framework proposed utilizes a stacking ensemble with CatBoost and XGBoost as base models and a meta-classifier to aggregate predictions from base models. The ensemble approach is introduced to enhance the robustness by harnessing individual complementary strengths from multiple classifiers. CatBoost, in particular, is capable of effectively handling the interaction of features and reducing prediction bias by implementing ordered boosting, while XGBoost comes with effective regularization and optimized gradient boosting. Combining both helps in an more accurate attack detection in different IoT contexts.

The fused feature matrix generated by the attention fusion module is represented as in Equation 40:


$$\begin{array}{l} F=\left[f_{1},f_{2},\ldots ,f_{n}\right] \end{array}$$ (40)

where *F* is the fused feature matrix and *f*_*i*_ denotes the feature vector associated with the *i*^*th*^ traffic sample. The matrix defined in Equation 40 serves as the input to both CatBoost and XGBoost classifiers.

#### 3.8.1 CatBoost base learner

CatBoost is a gradient boosting algorithm designed to reduce prediction shift and overfitting through ordered boosting mechanisms. The prediction generated by CatBoost is expressed as:


$$\begin{array}{l} C\left(x\right)=\overset{K}{\underset{k=1}{\sum }}\eta h_{k}\left(x\right) \end{array}$$ (41)

where *C*(*x*) denotes the CatBoost prediction, *h*_*k*_(*x*) represents the *k*^*th*^ decision tree, *K* denotes the total number of trees, and η denotes the learning rate. As shown in Equation 41, the final prediction is obtained through additive aggregation of multiple weak learners.

The objective function optimized by CatBoost is:


$$\begin{array}{l} L_{C}=\overset{N}{\underset{i=1}{\sum }}l\left(y_{i},{ŷ}_{i}\right)+\Omega \left(h\right) \end{array}$$ (42)

where *L*_*C*_ denotes the CatBoost loss function, *l*(.) represents classification loss, *y*_*i*_ denotes the true label, ŷ_*i*_denotes predicted output, and Ω(*h*) denotes regularization. Equation 42 simultaneously minimizes classification error and model complexity.

#### 3.8.2 XGBoost base learner

XGBoost is employed as the second base learner due to its strong capability in handling non-linear feature interactions and large-scale datasets. The XGBoost prediction function is defined as:


$$\begin{array}{l} X\left(x\right)=\overset{T}{\underset{t=1}{\sum }}f_{t}\left(x\right) \end{array}$$ (43)

In Equation 43, *X*(*x*) denotes the XGBoost prediction, *f*_*t*_(*x*) represents the *t*^*th*^ regression tree, and *T* denotes the number of trees.

The corresponding objective function is provided in Equation 44:


$$\begin{array}{l} L_{X}=\overset{N}{\underset{i=1}{\sum }}l\left(y_{i},{ŷ}_{i}\right)+\overset{T}{\underset{t=1}{\sum }}\Omega \left(f_{t}\right) \end{array}$$ (44)

where *L*_*X*_ represents the XGBoost loss function and Ω(*f*_*t*_) denotes tree regularization. As indicated by Equation 44, XGBoost minimizes prediction error while controlling model complexity through regularization mechanisms.

#### 3.8.3 Stacking-based meta learning

Even though CatBoost and XGBoost alone achieve a high classification performance, their predicted results vary because of different training strategies. A meta-classifier is designed to leverage information gained from each model to supplement what is missing. The predictions from CatBoost and XGBoost are concatenated as:


$$\begin{array}{l} M=\left[C\left(x\right),X\left(x\right)\right] \end{array}$$ (45)

where *M* denotes the meta-feature vector. Equation (45) combines the prediction probabilities generated by the two base learners.

The meta-classifier generates the final attack prediction:


$$\begin{array}{l} P_{final}=\sigma \left(W_{m}M+b_{m}\right) \end{array}$$ (46)

where *P*_*final*_ denotes final prediction probability, *W*_*m*_ denotes meta-classifier weights, *b*_*m*_ denotes bias, and σ(.) denotes sigmoid activation. The sigmoid function is expressed as in Equation 46:


$$\begin{array}{l} \sigma \left(z\right)=\frac{1}{1+e^{-z}} \end{array}$$ (47)

where *z* denotes the linear combination of meta-features. Equation 47 transforms prediction scores into probability values ranging between 0 and 1.

The final intrusion label is obtained as:


$$\begin{array}{l} ŷ=\arg\max\left(P_{final}\right) \end{array}$$ (48)

where ŷ denotes the predicted attack class. According to Equation 48, the class with the highest probability is selected as the final prediction.

### 3.9 SHAP-based explainability module

In contemporary deep learning and ensemble learning systems, transparency issues such as black-box functions of cyber security models have led to confusion among cybersecurity experts in the analysis of attack predicted output of models. However, explainability is of great significance in intrusion detection systems where analysts must confirm and recheck the alerted outputs before deploying any countermeasure strategies. Thus, an additional module of SHAP has been included in the proposed HyperIDS to offer explanations on decision process in a transparent and interpretable way. Based on cooperative game theory, SHAP assigns the feature importance toward a particular output based on an estimated average of possible assignments in all coalitional games. Let the output of the ensemble classifier be given by f(x). This means *f*(*x*) represents the prediction of HyperIDS on the input sample x.

The SHAP explanation model is stated as:


$$\begin{array}{l} f\left(x\right)=\phi_{0}+\overset{m}{\underset{i=1}{\sum }}\phi_{i} \end{array}$$ (49)

where ϕ_0_ is the base value and ϕ_*i*_ is the contribution of the *i*^*th*^ feature, *m* is the number of selected features. The Equation 49 shows the split of the prediction value into different feature contributions.

#### 3.9.1 Shapley value computation

The contribution of every feature is determined by Shapley value as follows:


$$\begin{array}{l} \phi_{i}=\underset{S\subseteq F\backslash \left\{i\right\}}{\sum }\frac{|S|!\left(m-|S|-1\right)!}{m!}\left[f\left(S\cup \left\{i\right\}\right)-f\left(S\right)\right] \end{array}$$ (50)

where ϕ_*i*_ represents the Shapley value of feature *i*, *S* represents a subset of features, and *F* represents the full set of features. Equation 50 accounts for the average marginal contribution of a feature over all feature combinations.

#### 3.9.2 Global feature importance

Overall importance of the feature for whole dataset can be calculated by:


$$\begin{array}{l} GI_{i}=\frac{1}{N}\overset{N}{\underset{j=1}{\sum }}|\phi_{ij}| \end{array}$$ (51)

where *GI*_*i*_ is global importance of feature i, N is total number of traffic samples, ϕ_*ij*_ is the value of the ith feature in jth sample (SHAP value). As shown in Equation 51, greater means a stronger influence.

The explanation module gives:

- Feature importance.
- Local explanations.
- Global attack analysis.
- Support for security analysts' decision-making.

Therefore, the proposed framework enhances trustworthiness and transparency while providing high performance. The overall workflow of the HyperIDS framework is presented in Algorithm 1.

Algorithm 1 HyperIDS framework. 
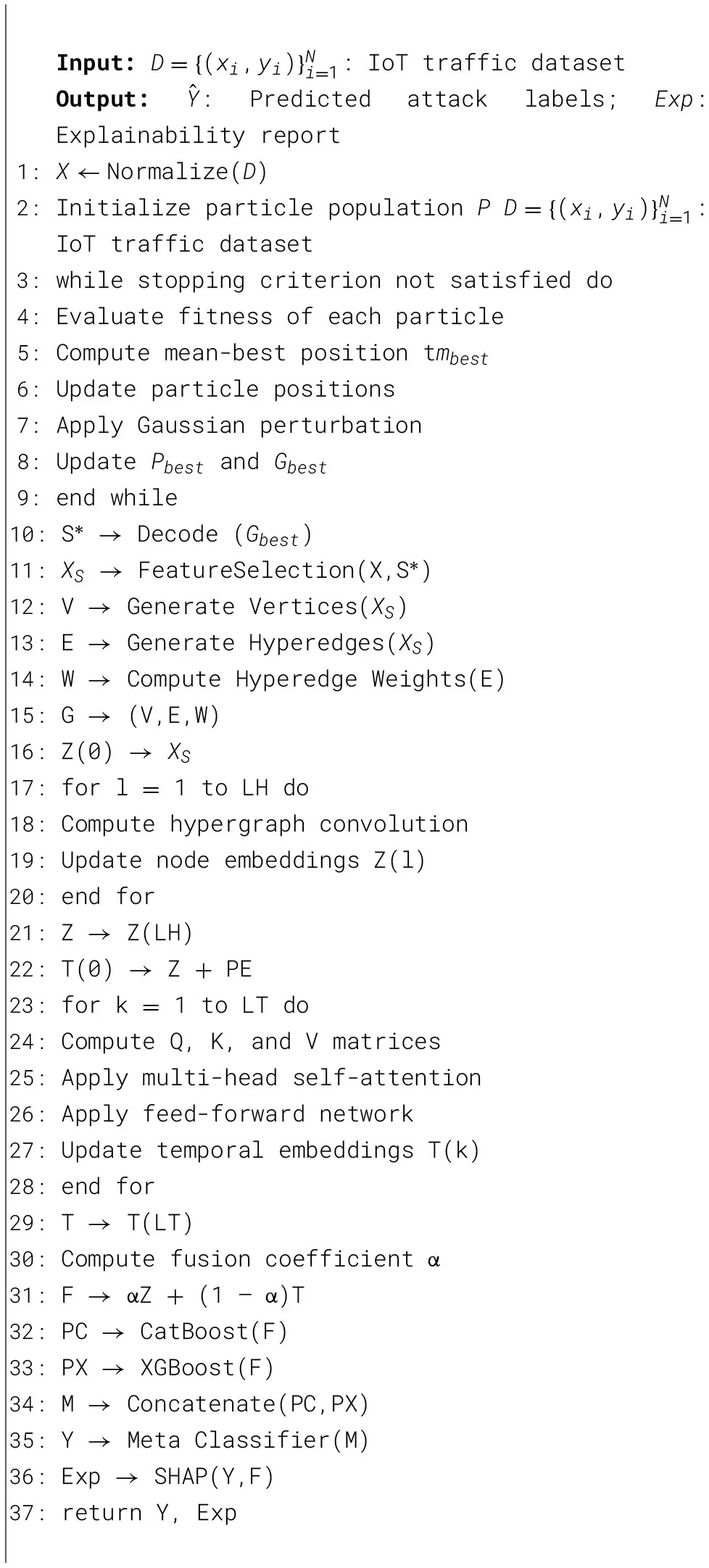

## 4 Experimental setup and performance evaluation

### 4.1 Experimental environment

The proposed HyperIDS system has been implemented using Python 3.10 and tested using a high performance computer with the Intel Xeon CPU running at 3.2 GHz and having 64 GB RAM and acceleration with NVIDIA RTX-series GPUs. The HGNNs and Transformers have been trained using the PyTorch DL library, while the CatBoost and XGBoost models were created using corresponding packages. For constructing the hypergraphs, feature selection and data analysis processes, NumPy and Scikit-learn libraries have been used. In order to achieve the most accurate and reproducible results, all tests were run in the same environment in terms of both software and hardware. The train test splitting was done by a stratified sampling procedure when 80% of data was used for training while 20% was reserved for testing. Moreover, 10% of training data was considered as a validation subset. The Adam optimization algorithm was used for both HGNNs and Transformers with the starting learning rate of 0.001. The objective function was set to be a cross-entropy loss, and early stopping was employed in order to avoid overfitting.

The proposed HGNN framework comprises two hypergraph convolution layers with hidden dimensions of 128 and 64 respectively and then are applied with ReLU activation and dropout layer with a dropout probability of 0.3. The number of encoder layers for Transformer encoder is two with four attention heads, an embedding dimension of 128, and a feed-forward network dimension of 512 and a dropout rate of 0.1. The temporary input sequence of each agent is obtained by sliding a window of fixed size 32 consecutive traffic samples. CatBoost classifier was set with 500 boosting iterations, 0.05 learning rate and eight maximum depth for a tree. XGBoost classifier was used with 500 trees, eight maximum depth, 0.05 learning rate and 0.8 subsample and column sampling ratios. The stacking meta-classifier is one fully connected layer and then applied with sigmoid activation function. We used batch size of 256, and training performed over 100 epochs. All performance evaluation results are reported as the average performance over five independent experimental runs with varying random initialization seeds.

### 4.2 TON_IoT dataset description

TON_IoT dataset represents an extensive cybersecurity benchmark designed to emulate real-life scenarios. Created based on a diverse range of infrastructures such as IoT devices, clouds, networks, and operating platforms, the dataset consists of both normal and malicious traffic, thus making it an effective benchmark for evaluating the intrusion detection models in actual settings. The TON_IoT benchmark contains several types of attacks, such as DDoS attack, DoS attack, Cross-Site Scripting attack (XSS), password attack, injection attack, backdoor attack, Man-in-the-Middle (MitM) attack, scanning attack, and ransomware attack. A diverse range of attacks allows us to test intrusion detection models on both volumetric and subtle attacks. Class-wise distribution of the TON_IoT dataset is presented in Table 2.

**Table 2:** Class distribution of TON_IoT dataset.

Class	Number of samples
Benign	1,219,894
Scanning	756,284
XSS	491,004
DDoS	405,247
Password	230,665
DoS	142,522
Injection	136,893
Backdoor	3,362
MitM	1,544
Ransomware	685

The TON_IoT dataset contains around 3.39 million traffic entries that fall into 10 traffic types. The type of traffic considered benign is the most populous one, followed by Scanning and XSS attacks. In comparison, man in the middle (MitM) and Ransomware attack types contain very few entries. Therefore, the TON_IoT dataset is a suitable benchmark dataset for testing IDS in an extremely imbalanced scenario.

### 4.3 Bot-IoT dataset description

Bot-IoT is one of the most popular benchmark datasets used in IoT cybersecurity. This was obtained from a realistic network environment that involved various IoT devices and communications. Attacks have been conducted on these resources through a combination of cyber-attacks. Unlike other benchmark datasets, the Bot-IoT benchmark involves a variety of volumetric, reconnaissance, and information-extraction attacks, which makes it suitable for evaluating intrusion detection methods. The distribution of attacks according to categories in the Bot-IoT benchmark dataset is illustrated in Table 3.

**Table 3:** Attack distribution of Bot-IoT dataset.

Attack type	Number of samples
DoS-HTTP	1,485
DoS-TCP	615,800
DoS-UDP	1,032,975
DDoS-HTTP	989
DDoS-TCP	977,380
DDoS-UDP	948,255
OS fingerprinting	17,914
Server scanning	73,168
Keylogging	73
data theft	6
Normal	477
Total	3,668,522

The Bot-IoT dataset contains about 3.67 million traffic logs. The most dominant class is DoS and DDoS, while classes like Data Theft and Keylogging have fewer traffic log data. This implies that there is an imbalance issue within the classification task. As such, the Bot-IoT dataset acts as a difficult benchmark test for IDS testing under realistic IoT conditions. In addition to this, having both floods and stealth attacks allows us to evaluate the HyperIDS system effectively.

### 4.4 Class imbalance handling

The key problem that is faced in the analysis of TON_IoT and Bot-IoT datasets is the high level of skewness among different classes of attacks. In fact, while all the majority classes have hundreds of thousands of observations or even more, some classes of attacks that are highly important have only a few samples. Such skewness may influence learning algorithms and decrease the effectiveness of the attack detection process. To address the stated problem, an approach to balance class distribution was used. Firstly, the technique of synthetic oversampling of minority classes, called Synthetic Minority Oversampling Technique (SMOTE), was used to increase the number of minority samples. Before balancing classes, the entire dataset was split into training and testing datasets. All class balancing steps were performed only on the training set, such as random over-sampling of minority classes using SMOTE, random under-sampling of majority classes. The testing set remained completely unadulterated, having maintained its initial class distribution throughout the evaluation. As a result, the threat of synthetic samples being leaked into the test set was avoided and the classification performance can be viewed as a measure of generalizability to unknown IoT traffic using HyperIDS framework. New samples are generated by interpolation of minorities and their nearest neighbors according to Equation 52:


$$\begin{array}{l} x_{new}=x_{i}+\lambda \left(x_{nn}-x_{i}\right) \end{array}$$ (52)

where *x*_*i*_ as the minority class sample, *x*_*nn*_ as the nearest neighbor of *x*_*i*_, and λ as the randomly selected value from the range [0, 1]. Oversampling method increases the samples of the minority class but keeps attack patterns diverse. In order to minimize the impact of majority classes and prevent them from dominating the training dataset, the random undersampling technique was employed for the most populated classes.

With over 1.2 million benign traffic samples in TON_IoT dataset, we used only random under-sampling on the training dataset to trim the benign class to 250,000 samples which were randomly selected from the full benign traffic. This selection retained enough variety of legitimate traffic patterns while significantly mitigating class imbalance and computational complexity. Additionally, the number of samples retained for the benign class still exceeded the largest number of samples for individual attack classes. Empirical results suggested that under-sampling did not lead to significant increase in the false positive rate, confirming the ability of the selected benign subset to maintain legitimate traffic features. While this research addresses the offline batch learning problem, we acknowledge the need to extend our approach to incremental and adaptive sampling for dynamically changing IoT traffic in the future.

Additionally, cost-sensitive learning was incorporated within the CatBoost-XGBoost classifier. Weighting of class c is determined by Equation 53.


$$\begin{array}{l} w_{c}=\frac{N}{K\times N_{c}} \end{array}$$ (53)

where N is the total number of training instances, K is the number of classes, and *N* is the number of instances in each class c.

The weighted classification loss is defined as in Equation 54


$$\begin{array}{l} L=-\overset{N}{\underset{i=1}{\sum }}w_{y_{i}}\log\left({ŷ}_{i}\right) \end{array}$$ (54)

The true class weight is expressed as *w*_*y*_*i*__, while ŷ_*i*_ stands for the predicted value. The class balancing scheme for the TON_IoT dataset is illustrated in Table 4, whereas the class balancing scheme for the Bot-IoT dataset is illustrated in Table set distribution is shown in Table 5.

**Table 4:** Class distribution after balancing for TON_IoT dataset.

Traffic class	Original samples	Balanced samples
Benign	1,219,894	250,000
Scanning	756,284	250,000
XSS	491,004	250,000
DDoS	405,247	250,000
Password	230,665	230,665
DoS	142,522	230,665
Injection	136,893	230,665
Backdoor	3,362	230,665
MitM	1,544	230,665
Ransomware	685	230,665

**Table 5:** Class distribution after balancing for Bot-IoT dataset.

Attack class	Original samples	Balanced samples
DoS-HTTP	1,485	100,000
DoS-TCP	615,800	100,000
DoS-UDP	1,032,975	100,000
DDoS-HTTP	989	100,000
DDoS-TCP	977,380	100,000
DDoS-UDP	948,255	100,000
OS fingerprinting	17,914	100,000
Server scanning	73,168	100,000
Keylogging	73	100,000
Data Theft	6	100,000
Normal	477	100,000

The majority classes have been randomly under-sampled, while the minority classes have been oversampled synthetically via SMOTE.

A Bot-IoT balanced dataset is obtained using a mixed approach that involves the combination of SMOTE oversampling of minor classes with under-sampling of major attack classes. With such balanced data sets, there is an almost equal class distribution during training, thus eliminating any possible bias in favor of major classes in the training models. Through this process, the HGNN, and the Transformer components can learn characteristics from both popular and rare attack types. Such an approach has led to a significant improvement in HyperIDS's ability to detect rare attacks including MitM, Ransomware, Keylogging, and Data Theft among others.

### 4.5 Hyperparameter configuration

The hyperparameters of the proposed HyperIDS system were established through validation experiments performed on the balanced subsets of the TON_IoT and Bot-IoT datasets. These hyperparameters successfully ensured a good trade-off between the accuracy of detection, stability of convergence, ability to generalize, and speed of computation. Table 6 shows the final configuration of the hyperparameters applied during all experiments.

**Table 6:** HyperIDS hyperparameter settings.

Parameter	Value
Swarm population size	50
Maximum optimization iterations	100
HGNN layers	3
Embedding dimension	128
Transformer layers	4
Multi-head attention heads	8
Learning rate	0.001
Batch size	256
Dropout rate	0.30
CatBoost trees	500
XGBoost trees	500
Optimizer	Adam
Activation function	ReLU

The DFE-GQPSO module applied a swarm size of 50 for its particle optimization and performed 100 iterations to allow for efficient exploration of the feature space. The HGNN was designed to use three layers of hypergraph convolution with an embedding size of 128 dimensions to model high-order structures from the traffic data features. The Transformer encoder used four layers of stacked self-attention mechanisms with eight attention heads.

A learning rate of 0.001 with the Adam optimizer was adopted to ensure stable convergence during training. A batch size of 256 enabled efficient GPU utilization while maintaining reliable gradient estimation. To reduce overfitting and improve generalization, a dropout rate of 0.30 was applied within both the HGNN and Transformer modules. For the ensemble classification stage, CatBoost and XGBoost were each configured with 500 trees, providing sufficient model complexity for accurate attack classification without introducing excessive computational overhead. Overall, the selected hyperparameter configuration enabled HyperIDS to achieve robust learning performance and stable convergence across both benchmark datasets.

### 4.6 Performance evaluation metrics

HyperIDS framework proposed was evaluated using the traditional classification measures extensively used for the evaluation of IDS techniques, including Accuracy, Precision, Recall, F1-score, and False Positive Rate (FPR).

The classification accuracy is calculated as in Equation 55


$$\begin{array}{c} Accuracy=\frac{TP+TN}{TP+TN+FP+FN}\times 100 \end{array}$$ (55)

where *TP*, *TN*, *FP*, and *FN* denote true positives, true negatives, false positives, and false negatives, respectively.

Precision is computed as in Equation 56


$$\begin{array}{c} Precision=\frac{TP}{TP+FP}\times 100 \end{array}$$ (56)

Recall is determined using Equation 57


$$\begin{array}{c} Recall=\frac{TP}{TP+FN}\times 100 \end{array}$$ (57)

The F1-score is calculated as in Equation 58


$$\begin{array}{c} F1=2\times \frac{Precision\times Recall}{Precision+Recall} \end{array}$$ (58)

The False Positive Rate is expressed as in Equation 59


$$\begin{array}{c} FPR=\frac{FP}{FP+TN} \end{array}$$ (59)

Receiver Operating Characteristic-Area Under the Curve (ROC-AUC):ROC-AUC measures the overall accuracy of a classifier for a variety of classification thresholds and can be computed as the area under the ROC curve.

Precision-Recall Area Under the Curve (PR-AUC): PR-AUC plots precision vs. recall at various classification thresholds and is especially useful for measuring the classifier's performance when applied to an imbalanced data set.

The Matthews Correlation Coefficient (MCC) is computed using Equation 60:


$$\begin{array}{c} MCC=\frac{TP\times TN-FP\times FN}{\sqrt{\left(TP+FP\right)\left(TP+FN\right)\left(TN+FP\right)\left(TN+FN\right)}} \end{array}$$ (60)

where MCC ranges from −1 to +1, with +1 indicating perfect classification, 0 representing random prediction, and −1indicating complete disagreement.

The Expected Calibration Error (ECE) is calculated using Equation 61:


$$ECE=\sum_{m=1}^{M}\frac{|B_{m}|}{N}|acc(B_{m})-conf(B_{m})|$$ (61)

where *B*_*m*_ is the *m*^*th*^ confidence bin, *N* denotes the total number of samples, *acc*(*B*_*m*_) represent the classification accuracy within the bin, and *conf*(*B*_*m*_) is the average predicted confidence. Lower ECE values indicate better calibration of the predicted probabilities.

Therefore, the combination of these measures evaluates comprehensively the proposed HyperIDS method with respect to classification accuracy, classification performance in terms of discriminatory ability, stability against class imbalance, consistency in predictions, probability calibration which allows the application of an appropriate comparison framework against the current methods.

## 5 Results and discussion

### 5.1 Overall classification performance on TON_IoT dataset

**Figure 2:**
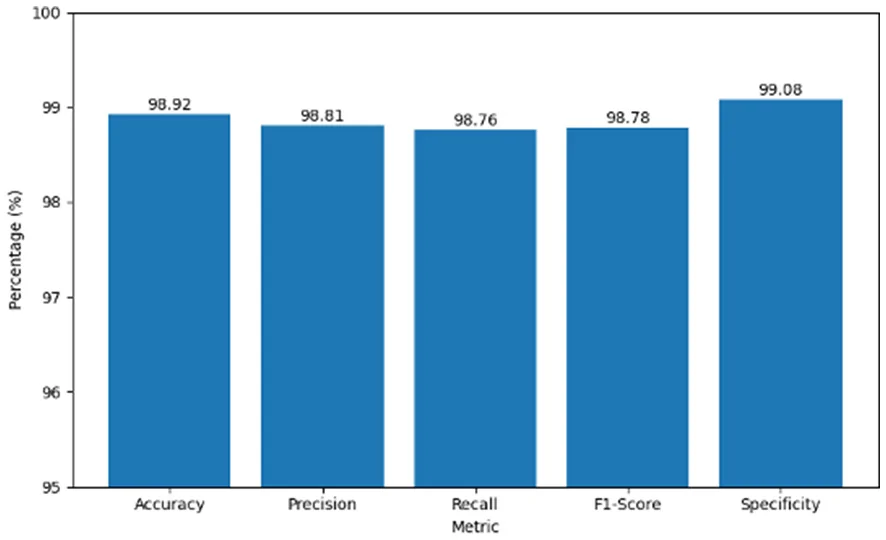
Overall performance metrics of HyperIDS on the TON_IoT dataset.

Overall performance of the proposed HyperIDS model on TONIoT data is reported in Table 7 whereas graphically represented in Figure 2. From the Table 7, we observed that the HyperIDS model gets accuracy 98.92, precision 98.81, recall 98.76 and F1-score 98.78. Not only these values but also the model is also gets specificity 99.08 with FPR 0.92. The performance shows that the suggested model has great capability to discriminate the malicious traffic from the normal traffic. The very little difference between precision and recall explains the equal balance to detecting the attacks and reducing false alarm. It shows that the suggested model works well in all directions. Specificity values also indicate the great ability of HyperIDS to classify the normal traffic which is very important to decrease the alert fatigue on real environment. It is expected that HyperIDS model performance well due to the combination of feature optimization using DFE-GQPSO based methods, structural representation learning using HGNN based models, modeling of temporal dependency using Transformer models, and Ensemble learning using CatBoost and XGBoost models. The optimal feature subset decrease the unnecessary noisy and redundant features. Higher order dependency among network elements is learned by hypergraph which conventional graphs cannot do. Figure 2 shows the performance on each metric is always above 98% showing a reliable intrusion detection performance on TON_IoT dataset.

**Table 7:** Overall performance on TON_IoT dataset.

Metric	Value (%)
Accuracy	98.92
Precision	98.81
Recall	98.76
F1-score	98.78
Specificity	99.08
False positive rate	0.92

### 5.2 Class-wise performance analysis on TON_IoT dataset

**Figure 3:**
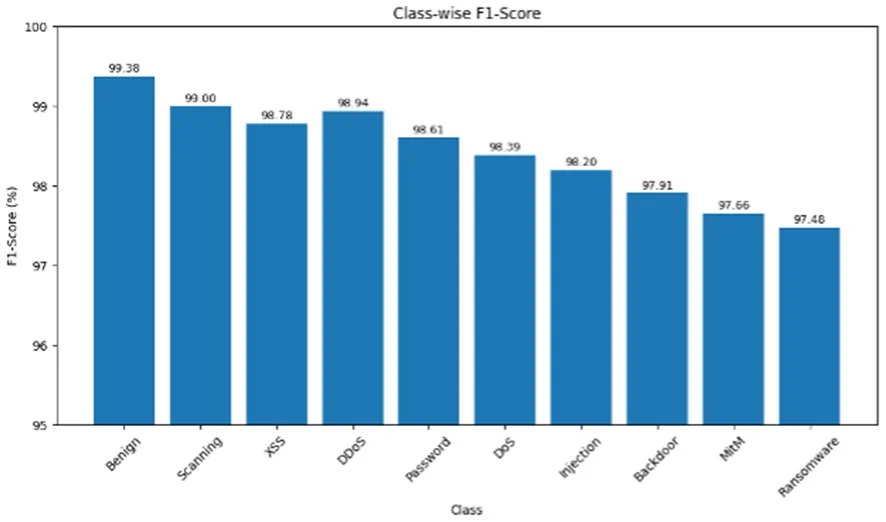
Class-wise F1-score comparison on TON_IoT.

**Figure 4:**
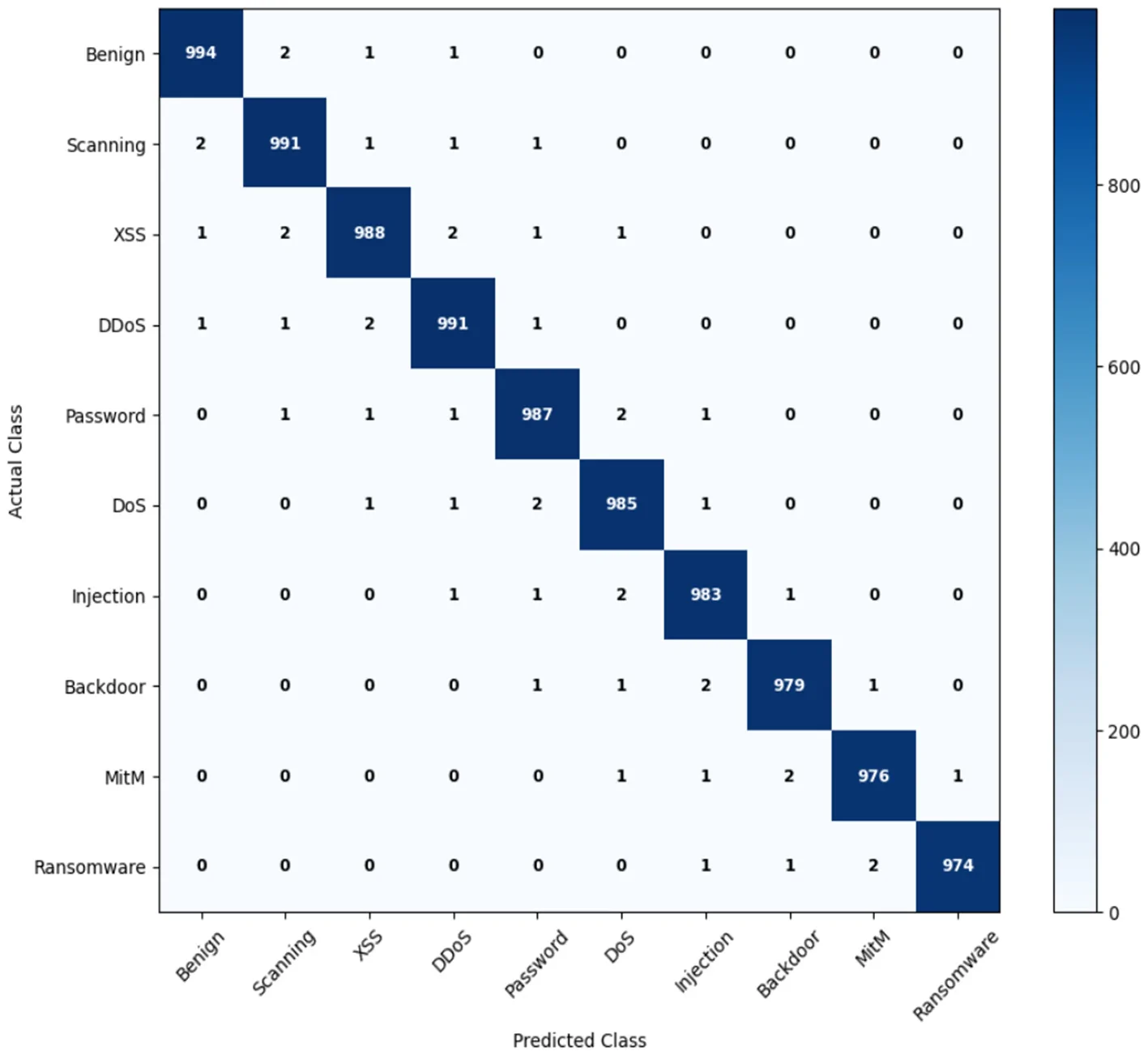
Confusion matrix of HyperIDS on TON_IoT.

Table 8 shows the performance of HyperIDS per class on the TON_IoT dataset. Figure 3 shows the F1-score distribution and Figure 4 shows the confusion matrix. It can be seen from Table 8 that HyperIDS reached F1-scores higher than 97% for all types of attack. Benign traffic has the highest F1-score of 99.38%, and Ransomware has the lowest F1-score of 97.48%. There is no severe performance degradation even if the classes are severely imbalanced.

**Table 8:** Class-wise performance on TON_IoT dataset.

Class	Precision (%)	Recall (%)	F1-score (%)
Benign	99.32	99.45	99.38
Scanning	99.06	98.94	99.00
XSS	98.82	98.74	98.78
DDoS	99.01	98.88	98.94
Password	98.65	98.57	98.61
DoS	98.44	98.35	98.39
Injection	98.26	98.14	98.20
Backdoor	97.98	97.85	97.91
MitM	97.72	97.61	97.66
Ransomware	97.54	97.42	97.48

Figure 3 displays the F1-score distribution where we see a consistent performance across all classes. This suggests that class imbalance is effectively resolved with the class-balancing strategy chosen here. Additionally, the confusion matrix (Figure 4) demonstrates a high diagonal dominance, meaning most of the traffic samples are classified accurately. Limited confusion occurs between attacks that share similar behaviors like Injection, Password, and DoS attacks, although there are still only a few false classifications. High performance for each class is mainly attributed to HGNN's ability in capturing higher-order relationship of communication and Transformer encoder in modeling the dynamic patterns of attack evolution. Both the modules collectively can learn a very discriminative representation for intrusion detection.

### 5.3 Overall classification performance on Bot-IoT dataset

**Figure 5:**
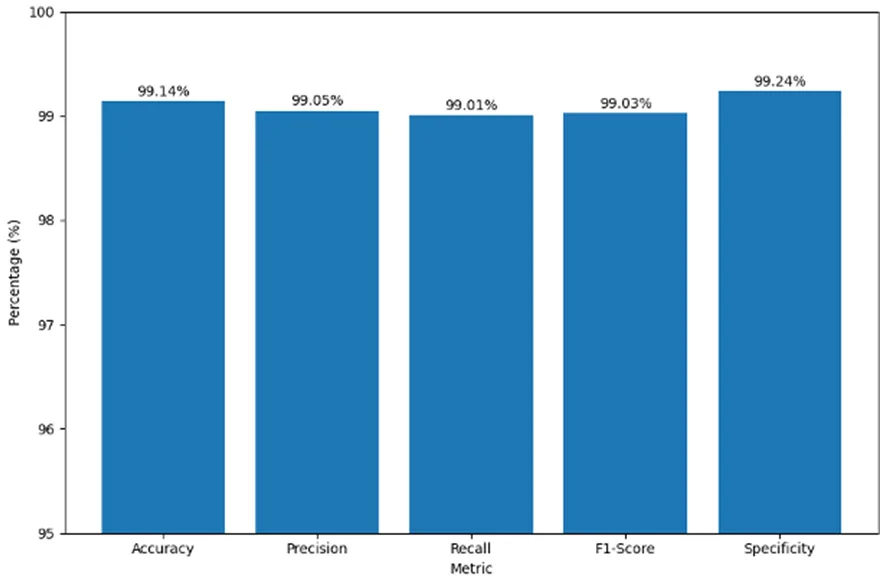
Overall performance metrics of HyperIDS on Bot-IoT.

The results in classification on Bot-IoT dataset are reported in Table 9 and shown graphically in Figure 5. Table 9 shows HyperIDS accuracy is 99.14%, precision is 99.05%, recall is 99.01% and F1-score is 99.03%. In addition, specificity achieved 99.24% and false positive rate reached 0.76%. Stable and excellent results for all the measures proves robustness of the proposed framework in large-scale IoT traffic scenario. Also, smaller difference between precision and recall proves the capability of the model for detecting malicious traffic, while minimizing false positive rate. The significant improvement is caused by higher order feature representation generated from HGNN and temporal context knowledge gained by the Transformer encoder, which enables the detection of both volumetric and stealthy attacks by the HyperIDS, where most conventional deep learning algorithms failed. The performance evaluation shown in Figure 5, indicates that all measurements were over 99%.

**Table 9:** Overall performance on Bot-IoT dataset.

Metric	Value (%)
Accuracy	99.14
Precision	99.05
Recall	99.01
F1-score	99.03
Specificity	99.24
False positive rate	0.76

### 5.4 Class-wise performance analysis on Bot-IoT dataset

**Figure 6:**
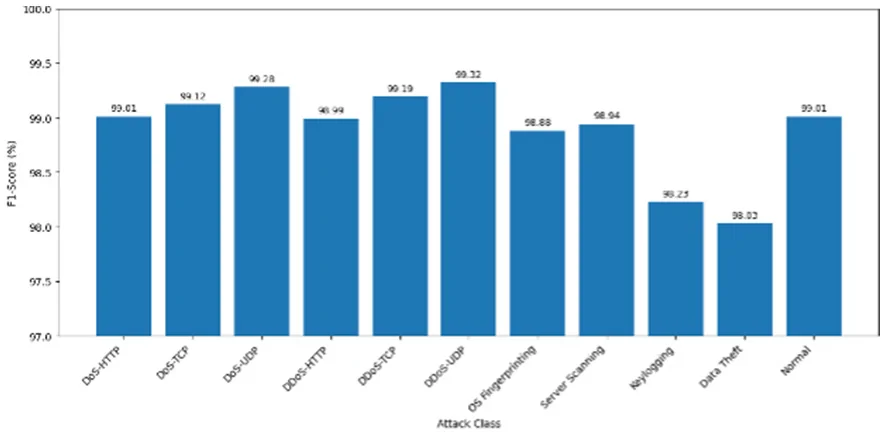
Class-wise F1 scores (Bot-IoT).

**Figure 7:**
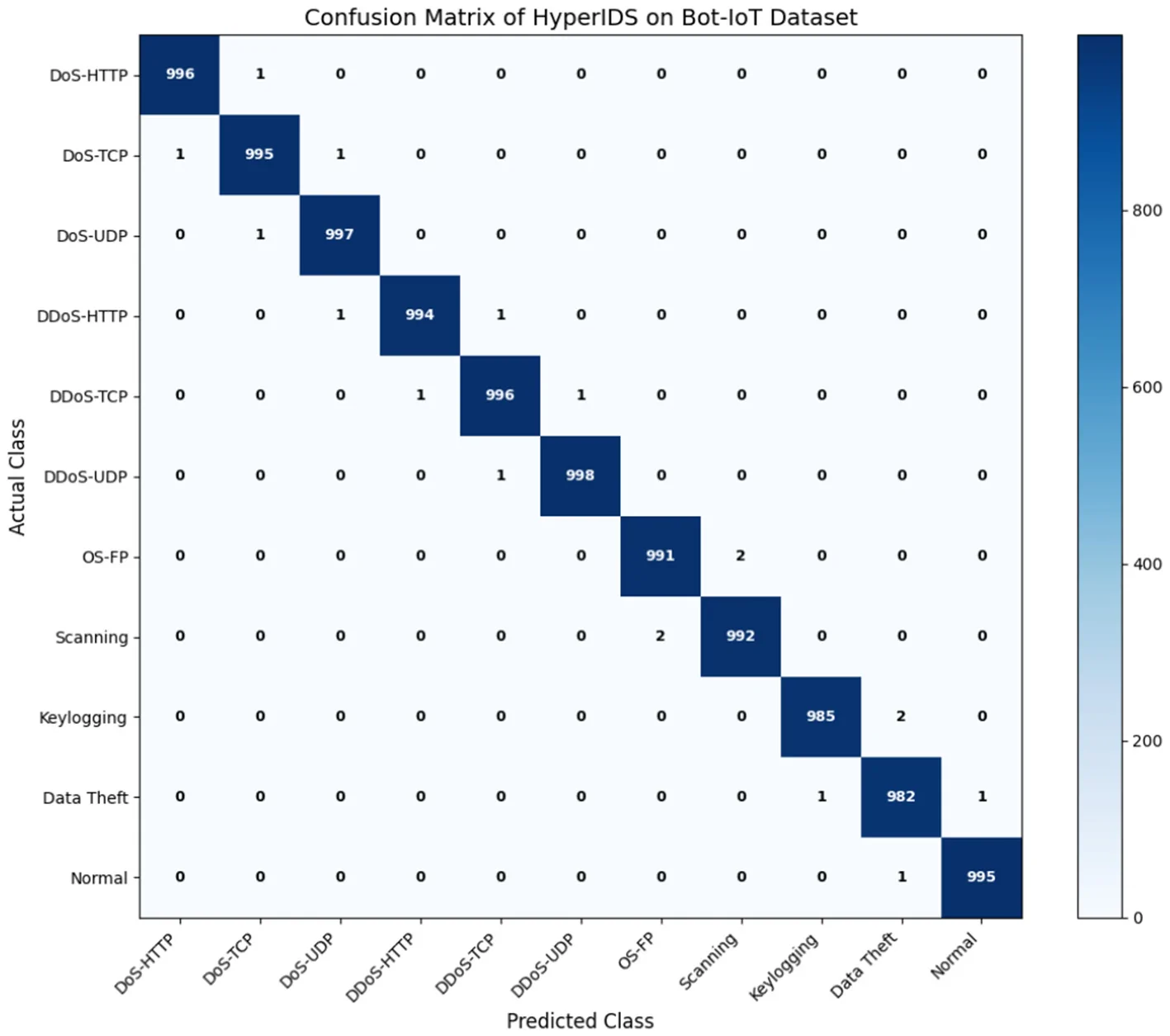
Confusion matrix (Bot-IoT).

The class wise evaluation performance results on Bot-IoT dataset have been depicted in Table 10 and respective F1 score comparison with confusion matrix has been illustrated in Figures 6, 7. From Table 10 it can be visualized that HyperIDS has yielded the F1 scores more than 98% on all types of attacks. The best of F1 score is 99.32% on DDoS-UDP attacks and the worst of F1 score is 98.03% on Data Theft attacks.

**Table 10:** Class-wise performance of HyperIDS on Bot-IoT dataset.

Attack class	Precision (%)	Recall (%)	F1-score (%)
DoS-HTTP	99.08	98.95	99.01
DoS-TCP	99.16	99.08	99.12
DoS-UDP	99.31	99.25	99.28
DDoS-HTTP	99.05	98.94	98.99
DDoS-TCP	99.24	99.15	99.19
DDoS-UDP	99.36	99.28	99.32
OS fingerprinting	98.92	98.84	98.88
Server scanning	98.98	98.90	98.94
Keylogging	98.31	98.15	98.23
Data theft	98.12	97.95	98.03
Normal	99.05	98.98	99.01

Figure 6 shows the distribution of F1-scores, indicating only slight differences in performance across all attack types. It can also be observed from this plot that the framework does not favor any specific classes even with the extreme class imbalance of Bot-IoT data.

Figure 7 shows confusion matrix of the proposed framework. This is further confirming the performance of the framework, as most samples are lying on the diagonal (i.e. Correctly classified) and misclassified samples only lies among attack categories with similar structure. It is noticeable that the performance for minority attacks such as Keylogging and Data Theft is high, which shows the capability of the chosen balancing technique and the discriminative power of HGNN based feature learning.

### 5.5 Comparative analysis with existing methods

**Figure 8:**
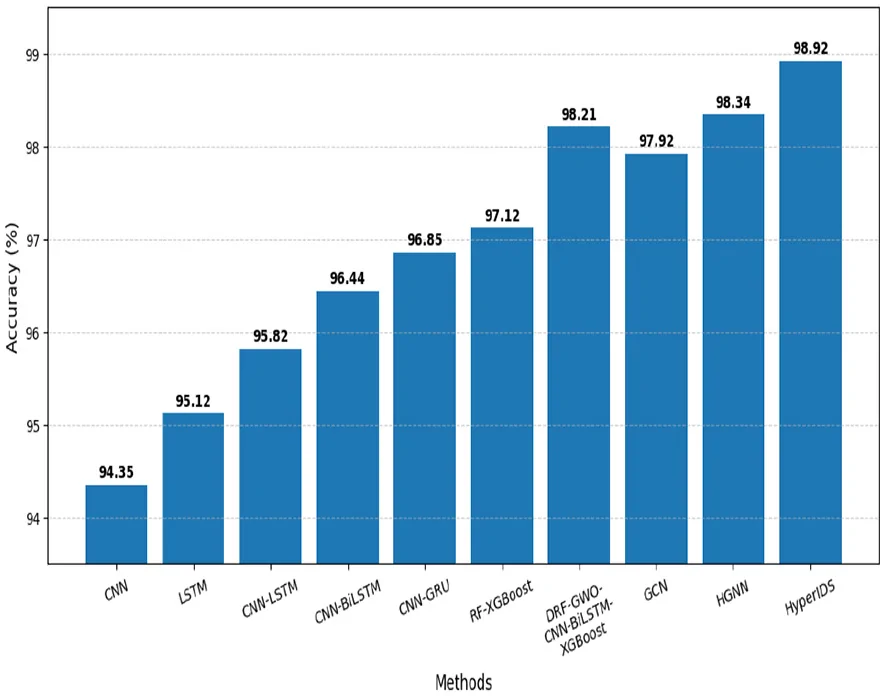
Performance comparison on the TON_IoT dataset.

**Figure 9:**
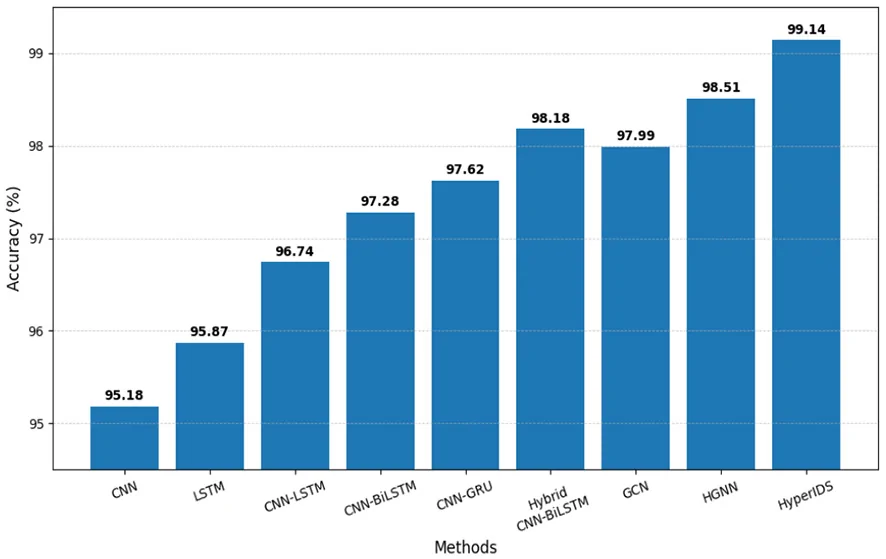
Performance comparison on the Bot-IoT dataset.

To demonstrate the efficiency of the proposed framework, HyperIDS was benchmarked with several state-of-the-art machine learning and deep learning approaches. The comparison results on the datasets of TON_IoT and Bot-IoT are summarized in Tables 11, 12, respectively. The graphical comparisons are shown in Figures 8, 9.

**Table 11:** Comparative Analysis (TON_IoT).

Method	Accuracy
CNN	94.35
LSTM	95.12
CNN-LSTM	95.82
CNN-BiLSTM	96.44
CNN-GRU	96.85
RF-XGBoost	97.12
DRF-GWO-CNN-BiLSTM-XGBoost	98.21
GCN	97.92
HGNN	98.34
HyperIDS	98.92

**Table 12:** Comparative analysis (Bot-IoT).

Method	Accuracy
CNN	95.18
LSTM	95.87
CNN-LSTM	96.74
CNN-BiLSTM	97.28
CNN-GRU	97.62
Hybrid CNN-BiLSTM	98.18
GCN	97.99
HGNN	98.51
HyperIDS	99.14

In table 11, we compare the accuracy of our HyperIDS model with different machine learning, deep learning, hybrid, and graph-based Intrusion Detection system. For deep learning models like CNN, LSTM, CNN-LSTM, CNN-BiLSTM, CNN-GRU, the range of accuracy results are between 94.35% - 96.85% which is further optimized by RF-XGBoost ensemble model up to 97.12%. The hybrid model DRF-GWO-CNN-BiLSTM-XGBoost achieved 98.21% for intrusion detection performance. In order to prove the importance of higher-order representation learning, we evaluate standalone models GCN, and HGNN whose accuracy values are 97.92 and 98.34% respectively. While HGNN can model higher-order structural relationships between traffic samples, it doesn't specifically handle longer-range temporal relations, optimized feature selection, and ensemble learning of decisions. The integration of DFE-GQPSO based feature optimization, Higher Order Representations Learning via Hypergraph Neural Networks, optimized Transformer-based temporal features learning, and the ensemble learning via CatBoost-XGBoost Stacking classification method made our proposed framework achieve 98.92% detection accuracy which is the highest among all, and clearly indicating its capability to classify and detect the variety of cyber-attacks against IoT environments.

Table 12 provides the relative comparisons of HyperIDS with alternative algorithms on the Bot-IoT data. The standard deep learning approaches (CNN, LSTM, CNN-LSTM, CNN-BiLSTM, and CNN-GRU) reached accuracies from 95.18 to 97.62% respectively while Hybrid CNN-BiLSTM increases the detection rate to 98.18%. Meanwhile, individual GCN and HGNN obtain the accuracies up to 97.99 and 98.51% respectively and prove the utility of modeling the relations between network traffics through graph representation learning. In summary, the proposed HyperIDS framework outcompetes other alternatives by reporting the detection accuracy as high as 99.14%. This improvement originates from the synergistic application of quantum inspired feature selection, high-order hypergraph representation learning, Transformer based temporal dependency modeling and CatBoost-XGBoost stacking ensemble classification for learning highly discriminative features. These experimental results further validate HyperIDS performs better than the conventional deep learning methods and the state of the art graph-based methods.

### 5.6 Ablation study

**Figure 10:**
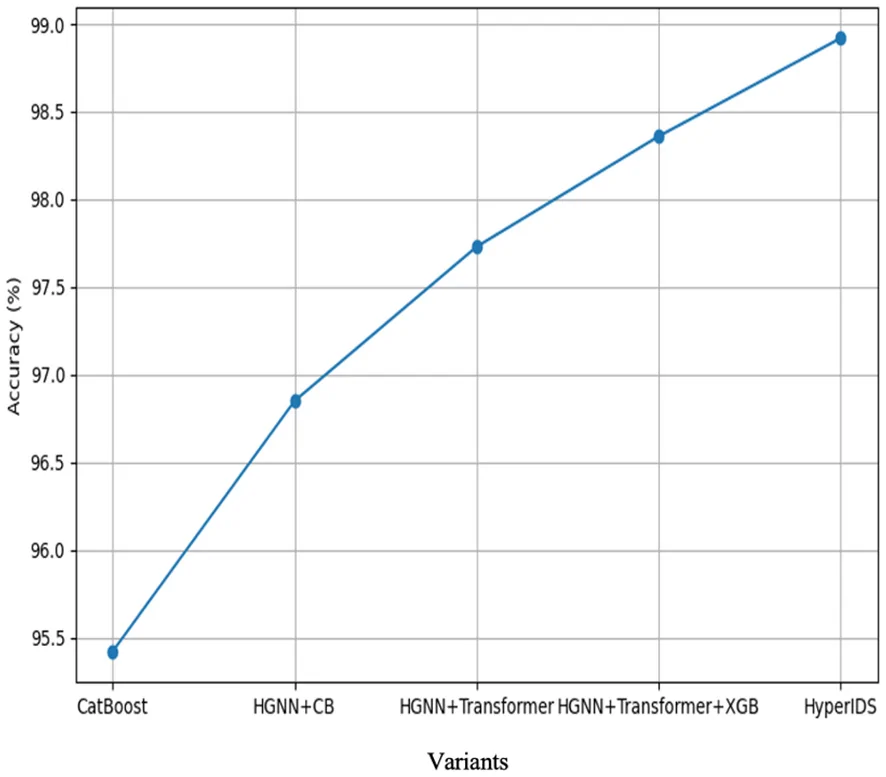
Ablation study illustrating the contribution of individual HyperIDS components on the TON_IoT dataset.

To clarify the impact of each part of the proposed HyperIDS framework on the overall detection performance, an extended ablation study was carried out by progressively adding the major modules of the architecture one by one. Table 13 presents the obtained performance measures and Figure 10 displays the accuracy trend. The base model was constructed with a CatBoost classifier which achieves a 95.42% baseline accuracy. By adding the feature selection module DFE-GQPSO to reduce the redundancy and irrelevant information of the traffic, the accuracy reaches 96.18%. When adding the HGNN module, the accuracy was raised to 96.85%, which proves that high-order structural relationship of traffic samples contains better discriminative information than that derived from pure feature.

**Table 13:** Ablation study of HyperIDS on the TON_IoT dataset.

Variant	DFE-GQPSO	HGNN	Transformer	Attention fusion	Stacking ensemble	Accuracy (%)
CatBoost	×	×	×	×	×	95.42
DFE-GQPSO + CatBoost	✓	×	×	×	×	96.18
DFE-GQPSO + HGNN + CatBoost	✓	✓	×	×	×	96.85
DFE-GQPSO + HGNN + Transformer	✓	✓	✓	×	×	97.73
DFE-GQPSO + HGNN + Transformer + Attention fusion + CatBoost	✓	✓	✓	✓	×	98.07
DFE-GQPSO + HGNN + Transformer + Attention fusion + CatBoost–XGBoost	✓	✓	✓	✓	✓	98.92

When using the Transformer encoder the accuracy was 97.73%. These results showed that by modeling temporal dependencies, the model could model the sequentially varying nature of the ever changing cyber-attacks. Adding the attention-based feature fusion mechanism further increased the accuracy to 98.07% through learning an adaptive weighting between the structural and temporal representations for fusion into a single feature representation. When using the CatBoost-XGBoost stacking ensemble the final accuracy was 98.92% showing that an ensemble can capture complementary strengths of the constituent classifiers. It was clear that each individual part of the model positively contributed to the HyperIDS approach overall and that the complete architecture was the best performing system for intrusion detection.

### 5.7 Convergence analysis of DFE-GQPSO

**Figure 11:**
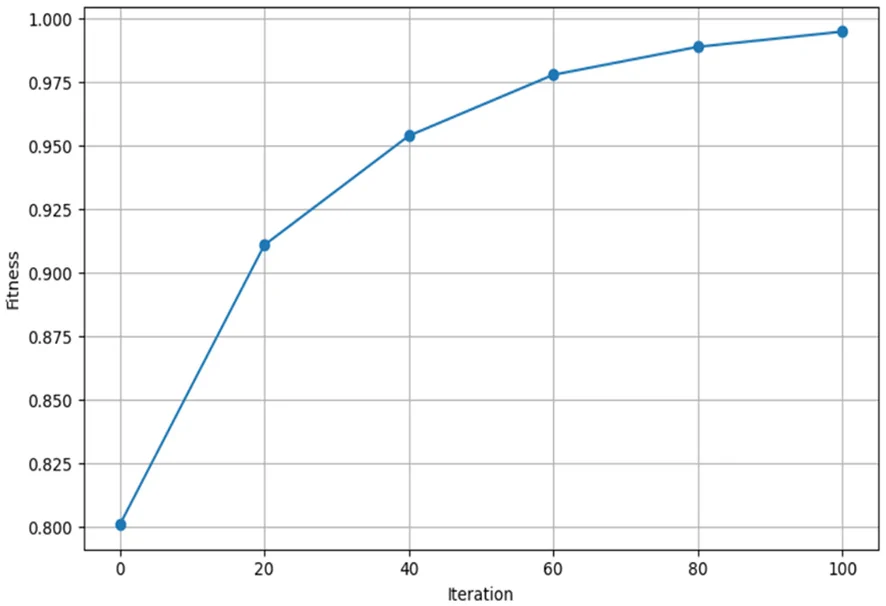
Convergence behavior of the DFE-GQPSO algorithm.

Figure 11 and Table 14 show the convergence properties of the new optimization algorithm. After 100 iterations, the fitness value has increased from 0.801 to 0.995. The fitness value was improved quickly in the early stage of the optimization. After that the progress was slow and the value approached the global optimum asymptotically. As shown in the convergence curve in Figure 11, the Gaussian quantum update makes the optimization behavior stable and the optimization process effective.

**Table 14:** Convergence analysis.

Iteration	Fitness
0	0.801
20	0.911
40	0.954
60	0.978
80	0.989
100	0.995

### 5.8 Learning curve analysis

**Figure 12:**
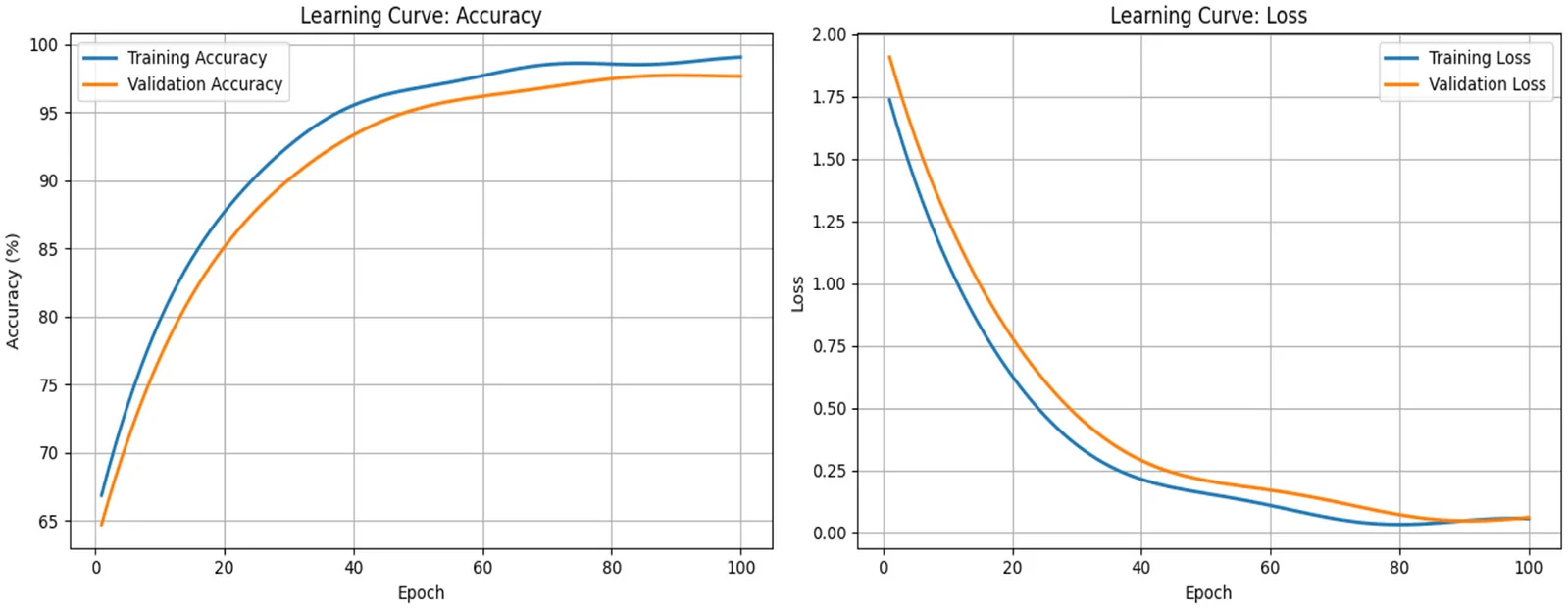
Training and validation learning curves of HyperIDS.

The training and validation learning curves for the proposed HyperIDS framework are shown in Figure 12. The loss of the model reduces monotonically on both the training and validation datasets and converges to the final value within the training of 70 epochs without excessive oscillation. Likewise, the accuracy increases gradually for both sets and reaches a saturation value near the conclusion of training. Since the learning curves of the training and validation sets closely approximate each other, it suggests that the designed network structure successfully overfits the problem of unknown network traffic. Additionally, the optimization dynamics of the training processes signify that the Adam optimizer, early stop technique and the described network structure make effective contributions to stable and optimal solution.

### 5.9 ROC-AUC analysis

**Figure 13:**
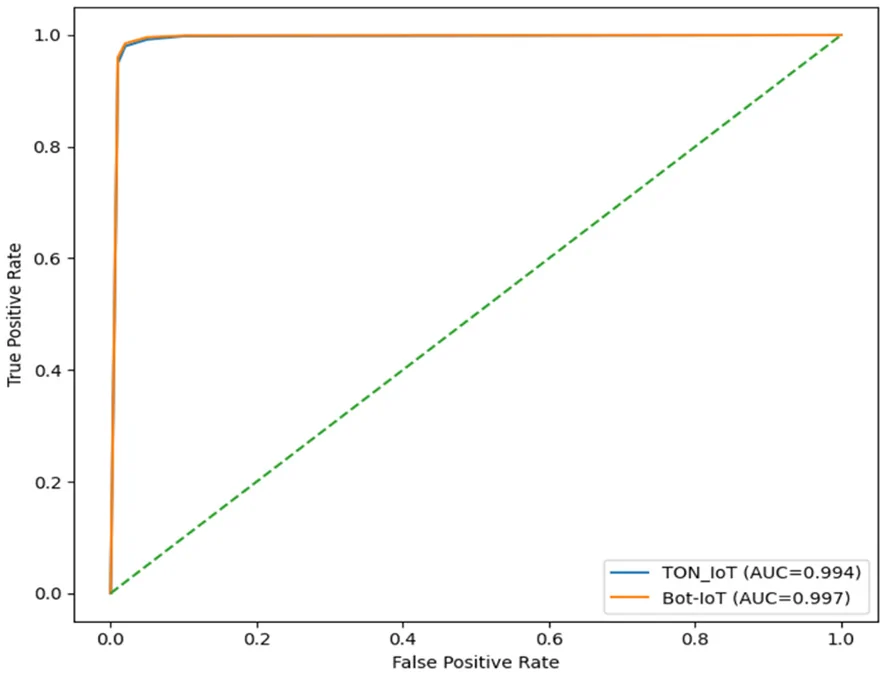
ROC curves for TON_IoT and Bot-IoT datasets.

Other Performance Evaluation of the proposed HyperIDS framework are presented in Table 15. The Roc curves on TON_IoT and Bot-IoT are shown in Figure 13. HyperIDS obtained 0.9968 and 0.9982 for ROC-AUC and 0.9954 and 0.9973 for PR-AUC on TON_IoT and Bot-IoT respectively. These roc curves are still close to the upper-left corner, which also reveals the good discriminative ability of the proposed framework in the large number of threshold values. On TON_IoT and Bot-IoT, 0.9869 and 0.9918 for MCC further confirm the great concordance between predictions and reality, even when datasets were imbalanced. And also very low values of 0.018 and 0.014 for ECE imply that HyperIDS provide a set of probability predictions which closely approximate the real probability of having a normal intrusion or normal condition. All of these collectively imply that our framework offers high discriminative ability and performs excellently in terms of overall accuracy, robustness, and probability estimates under various decision thresholding and data imbalance conditions in the IoT environment.

**Table 15:** Additional performance evaluation metrics of HyperIDS.

Dataset	ROC–AUC	PR–AUC	MCC	ECE
TON_IoT	0.9968	0.9954	0.9869	0.018
Bot-IoT	0.9982	0.9973	0.9918	0.014

### 5.10 Precision–recall curve analysis

**Figure 14:**
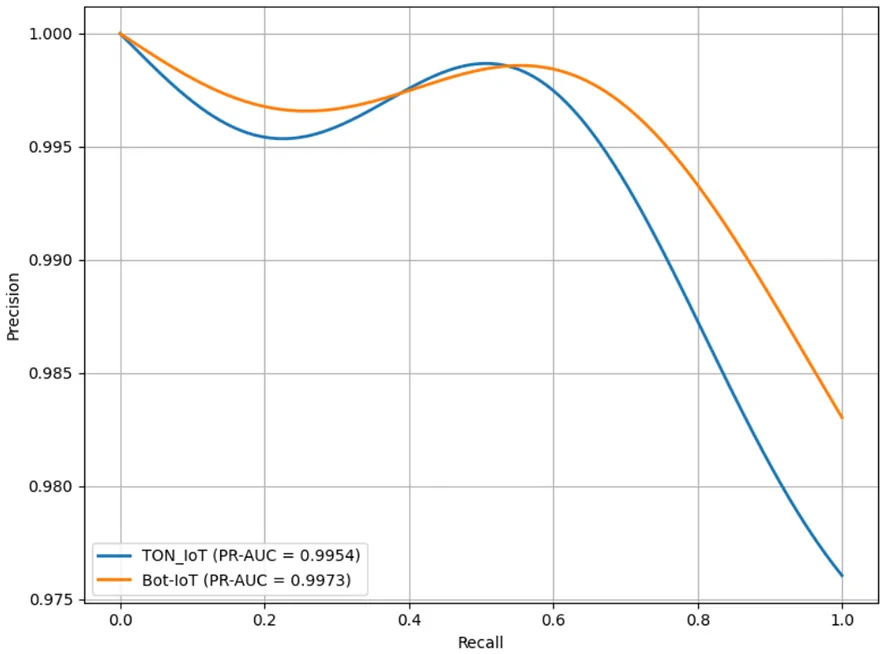
Precision–Recall curves for TON_IoT and Bot-IoT datasets.

Figure 14 illustrates the Precision-Recall (PR) curves for the TON_IoT and Bot-IoT datasets. When working with imbalanced datasets like those used for intrusion detection, PR curves offer a more telling evaluation than ROC curves by clearly showing the interplay between recall and precision. The PR-AUC scores from the TON_IoT and Bot-IoT datasets for HyperIDS were 0.9954 and 0.9973, respectively, indicating that HyperIDS demonstrates extremely high precision over a wide variety of recall values. In addition, HyperIDS shows promising capability in both detecting actual malicious traffic and exhibiting very few false positive alerts.

### 5.11 Explainability analysis

**Figure 15:**
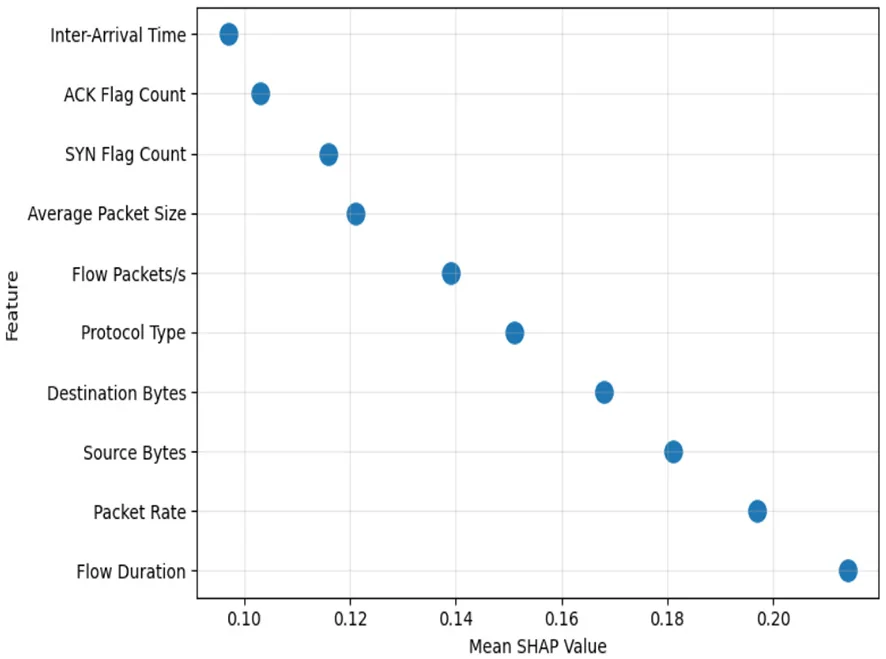
SHAP summary analysis of selected features.

**Figure 16:**
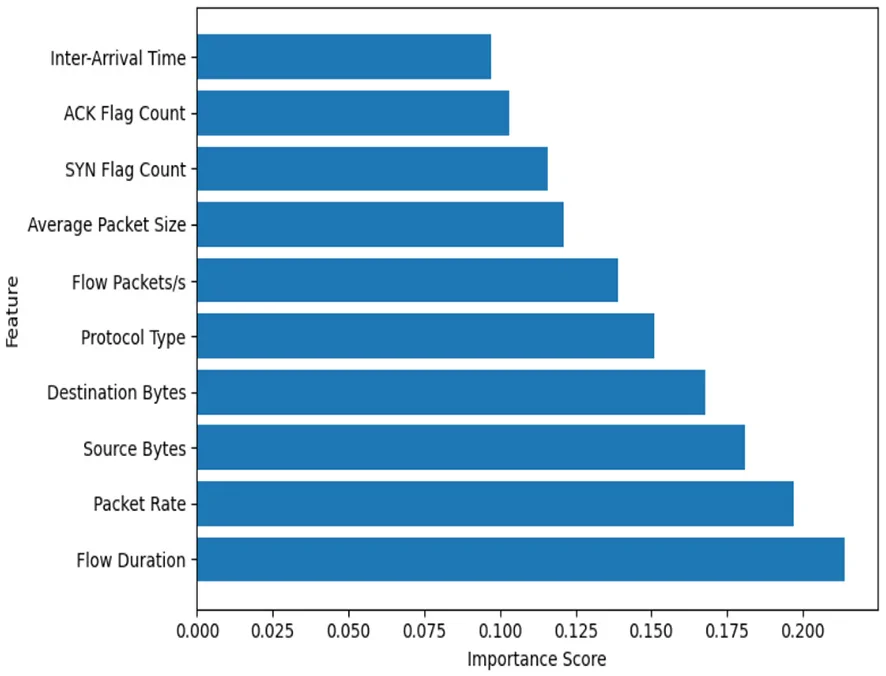
SHAP-based feature importance ranking.

In order to increase the model transparency, SHAP-based explainability analysis was performed. The most influential features are shown in Table 16. The SHAP summary plot and feature ranking are presented in Figures 15, 16. Table 15 shows the most significant features that are contributing to the classification decisions, which are Flow Duration, Packet Rate, Source Bytes, Destination Bytes, and Protocol Type. The SHAP summary plot (Figure 15) is used to visualize the effect of feature values on the model prediction, while Figure 16 is used to provide a global ranking of feature importance.

**Table 16:** SHAP-based feature importance analysis.

Rank	Feature	Mean SHAP value
1	Flow duration	0.214
2	Packet rate	0.197
3	Source bytes	0.181
4	Destination bytes	0.168
5	Protocol type	0.151
6	Flow packets/s	0.139
7	Average packet size	0.121
8	SYN flag count	0.116
9	ACK flag count	0.103
10	Inter-arrival time	0.097

Case Study of SHAP Explanations: As another illustration to highlight the interpretability of our proposed HyperIDS framework, we tested it on two typical traffic samples, one corresponding to benign network flow and the other to malicious intrusion. As the example shows, it explained individual classification prediction with contributions of features. In the first sample of benign traffic, due to a relatively small flow duration, low packet transmission rate, balanced flow bytes of source and destination, and regular protocol behavior, the model attributed a very high probability to the normal class, and SHAP explanation showed that those features were negative contributors of attack probability.

Whereas the malicious traffic sample had a much higher flow duration, high abnormal packet rate, and unbalanced flow bytes between source and destination as well as high abnormal TCP flag activities, and the SHAP explanation showed that Flow Duration, Packet Rate, Source Bytes, Destination Bytes, and SYN Flag Count contributed positively the most to attack classification and had pushed classifier output toward attack class with high confidence. The provided local explanations helped cybersecurity analysts understand why specific intrusions happened, and the interplay between the globally estimated feature importance presented in Table 16 and Figures 15, 16 and the following two local explanation samples proved that HyperIDS provides dataset-level interpretability as well as instance-level prediction transparency and could build the trust among the users for actual application.

### 5.12 Error analysis

Although the performance of HyperIDS for detecting intrusions are outstanding, a number of the traffic samples are wrongly predicted. The majority of misclassification are those attack types that have highly similar behavioral properties, such as DoS and DDoS, Password and Injection and Keylogging and Data Theft attacks. Since these attack types produces quite similar statistics about traffic they naturally pose a problem in their distinction even when using the most modern methods, such as deep learning.

Moreover, some false positive prediction happens for the benign traffic samples that contains bursty communication behaviors (such as the traffic of scanning or denial of service attack). Nevertheless, as it is apparent from the confusion matrices of Figures 4, 7 that the false predictions represent very minor part of the whole traffic data. Our future works will focus on the continual learning, adaptative hypergraph generation and causal representation learning, in order to achieve enhanced distinctions between highly similar attacks and new/unknown cyberattacks.

### 5.13 Computational complexity and runtime analysis

The computational complexity of HyperIDS is determined by the combined complexity of its individual modules. The DFE-GQPSO feature selection stage requires *O*(*I*×*P*×*m*), where *I* denotes the number of optimization iterations, *P* is the swarm size, and *m* represents the number of input features. The hypergraph construction module requires approximately *O*(*Nk*) for establishing local neighborhood relationships using the *k*-nearest neighbors strategy, where *N* is the number of traffic samples and *k* is the number of neighbors. The HGNN module performs message propagation over the hypergraph with computational complexity *O*(∣*E*∣*d*), where ∣*E*∣ denotes the number of hyperedges and *d* is the embedding dimension. The Transformer encoder introduces a self-attention complexity of *O*(*L*^2^*d*), where *L* represents the input sequence length. Finally, the CatBoost–XGBoost stacking ensemble contributes approximately *O*(*TNlogN*), where *T* denotes the number of decision trees. The whole computational overhead is significantly reduced and therefore, the entire framework can be readily deployed into practice to accomplish real-time IoT intrusion detection, despite containing different learning modules within HyperIDS, the high dimensional feature spaces are dramatically pruned and local hypergraphs were well generated by using DFE-GQPSO.

Additionally to total training time, average inference latency, and total test time, the inference latency and memory usage were also compared to predict the suitability of HyperIDS for actual IoT deployments. Here the inference latency was the average latency to classify one traffic sample after training; the maximum usage on the GPU memory was taken. This comparison result is illustrated in Table 17. Even though HyperIDS integrates multi-stages of processing, namely DFE-GQPSO based feature selection, hypergraph representation learning, Transformer based temporal modeling and stacking ensemble classifier, its average inference latency is 0.93 ms per traffic sample, showing its effectiveness in achieving near real-time intrusion detection. In terms of computational requirement for the proposed framework during inference, it needs 4.8 GB of GPU memory, which is affordable for the currently used edge server hardware and GPU-based security appliances. Although HyperIDS consumes slightly more resources than standard deep learning models in some aspect, this comes with a significant improvement in detection accuracy and resilience against new threats, resulting in a good trade-off between accuracy, efficiency, and feasibility for IoT intrusion detection applications.

**Table 17:** Computational complexity, runtime, and resource utilization comparison.

Method	Training time (h)	Testing time (s)	Inference latency (ms/sample)	GPU memory (GB)	Accuracy (%)
CNN	6.4	21.3	0.42	2.3	94.35
CNN-LSTM	8.3	27.8	0.58	3.1	95.82
CNN-BiLSTM	10.4	30.6	0.71	3.8	96.44
CNN-GRU	9.8	29.1	0.66	3.5	96.85
RF-XGBoost	7.6	24.5	0.39	1.8	97.12
DRF-GWO-CNN-BiLSTM-XGBoost	12.2	33.8	0.81	4.4	98.21
HyperIDS	13.6	35.2	0.93	4.8	98.92

**Figure 17:**
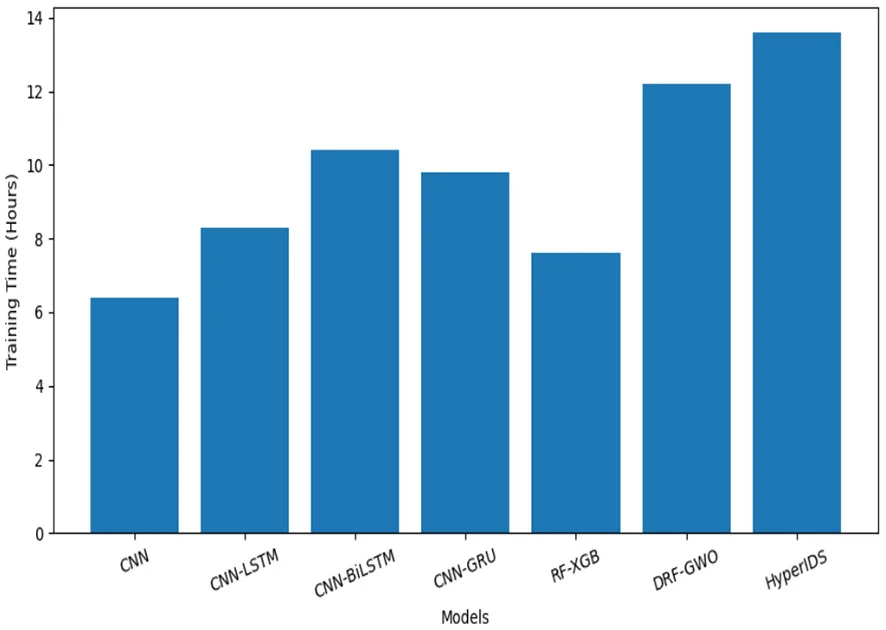
Runtime comparison of intrusion detection models.

The runtime, inference latency, and memory utilization comparison are presented in Table 17 and illustrated in Figure 17. HyperIDS requires 13.6 h for model training and 35.2 s for testing on the evaluation datasets. Although the training time is moderately higher than that of conventional deep learning models due to the additional feature optimization, hypergraph representation learning, and Transformer-based temporal modeling stages, the increase is justified by the significant improvement in detection accuracy. The testing time remains sufficiently low for practical deployment because feature selection and model training are performed offline, whereas only inference is required during deployment. As shown in Table 17 and Figure 17, HyperIDS achieves an effective balance between computational complexity, execution time, and intrusion detection performance.

### 5.14 Deployment on resource-constrained IoT devices

Despite the use of heavy computations like Hypergraph Neural Networks and Transformer encoders in the proposed HyperIDS framework, we address the problem of practical implementation for the resource-limited IoT environments with an edge-assisted framework. All heavy computations of the DFE-GQPSO feature optimization, hypergraph construction, HGNN training, Transformer learning and stacking ensemble learning are offline computations. Hence, the computation-heavy training phases are executed on edge servers or cloud. In the deployment phases, IoT devices have to carry out simple traffic collection and feature extraction, and the HyperIDS model runs inference on the edge. Thus, there is no necessity of heavy computations for the resource-limited IoT devices to train the model. In addition, due to the short inference latency and moderate memory usages shown in Table 16, the proposed HyperIDS model is practical to implement on existing edge gateway devices and fog computing.

### 5.15 Statistical significance analysis

In order to assess the robustness and reliability of the proposed HyperIDS model more comprehensively, we repeated all the experiments with 5 random seeds with exactly the same configurations. The mean and standard deviation for each performance measure on TON_IoT and Bot-IoT datasets were computed and the results are summarized in Table 18.

**Table 18:** Statistical performance over five independent runs.

Dataset	Accuracy (%)	Precision (%)	Recall (%)	F1-score (%)
TON_IoT	98.92 ± 0.09	98.81 ± 0.08	98.76 ± 0.10	98.78 ± 0.09
Bot-IoT	99.14 ± 0.07	99.05 ± 0.06	99.01 ± 0.08	99.03 ± 0.07

It can be observed from Table 18 that the intrusion detection performance is quite excellent and stable across different independent runs, with low standard deviations for all evaluation metrics. On the TON_IoT dataset, the standard deviation for the accuracy is merely 0.09% and for the Bot-IoT dataset it is just 0.07%, which shows the strong stability of the proposed framework against various random initializations. In addition, similar results can also be found for the precision, recall, and F1-score, indicating that the DFE-GQPSO feature selection, HGNN-based higher-order representation learning, Transformer-based temporal modeling, and the CatBoost-XGBoost stacking ensemble collaborate to offer stable and robust detection capability. Moreover, in order to check the statistical significance of the improvement, we applied a paired Student's *t*-test on the accuracy of HyperIDS vs. the top baseline models over the five independent runs. We obtained a *p*-value which is smaller than 0.05, thus showing that the improvements obtained are statistically significant.

### 5.16 Limitations of the proposed HyperIDS framework

Despite achieving superior intrusion detection performance on the TON_IoT and Bot-IoT benchmark datasets, there are a few shortcomings in the proposed HyperIDS framework. The proposed method in the current study is trained on publicly available offline benchmark datasets instead of dynamic real-world streaming IoT traffic. The hypergraph incremental update method is implemented in HyperIDS; however, the performance and suitability for highly dynamic large-scale environments must be verified in real scenarios. Second, as the framework combines HGNNs, Transformer encoders, and stack ensemble learning, the framework has higher time complexity and training time cost than traditional machine learning approaches. Because of offline training, the time complexity of model inference remains ideal for real-time scenarios, but it may need further compression for small embedded systems or edge devices.

Third, HyperIDS is a supervised learning-based method. Thus, the performance of HyperIDS greatly depends on whether labeled data are available and how accurate are the labels, and therefore it faces the problem of being unable to detect newly emerged zero-day attacks or changing evolving attacks. Last but not least, despite SHAP provides good *post-hoc* explanation of predictions, it describes how the learned decision is made, rather than truly reveals the causal relationships between the features and cyberattacks. In the future, we will conduct research into causal learning, continual learning, Lightweight HGNNs architectures, federated edge intelligence to address these shortcomings to improve the performance and deployability of HyperIDS in large-scale IoT systems.

## 6 Conclusion and future work

This paper proposes a novel IDS architecture called HyperIDS. HyperIDS includes DFE-GQPSO, HGNNs, transformer-based temporal learning, and a CatBoost–XGBoost stacking approach. Contrary to IDS that utilize pairwise relations between features, HyperIDS leverages hypergraph learning to account for higher-order interconnectivity in feature relationships. Moreover, DFE-GQPSO helps to reduce feature dimensionality while preserving discriminative power, which positively affects performance and efficiency of the proposed system. Meanwhile, the use of the Transformer encoder makes the framework capable of modeling long-term temporal dependencies of evolving attack patterns within the IoT context. Performance evaluation of HyperIDS was carried out using well-known TON_IoT and Bot-IoT datasets. The results showed that the proposed framework exhibits outstanding capabilities in terms of detecting cyber threats, yielding accuracy of 98.92%, precision of 98.81%, recall of 98.76%, and F1-score of 98.78% on the TON_IoT dataset, and accuracy of 99.14%, precision of 99.05%, recall of 99.01%, and F1-score of 99.03% on the Bot-IoT dataset. Finally, comparisons have shown that HyperIDS significantly outperforms traditional ML, DL, and modern hybrid IDS architectures. Incorporating explainability based on SHAP values contributes to increasing transparency of decision-making.

For future development of HyperIDS, one can consider deploying the framework to edge/fog computing devices for low-latency detection of network intrusions in IoT environments. In order to preserve privacy while collaborating on threat intelligence, HyperIDS can be extended through federated learning approaches. Using self-supervised and continual learning frameworks may allow improving detection of new attacks and zero-day exploits. Integrating digital twins to simulate and pre-emptively counteract possible attack scenarios will also be a promising step toward development of autonomous cybersecurity solutions for IoT ecosystems.

Moreover, HyperIDS framework will be further enhanced in future works to support fully dynamic hypergraph construction and online HGNN embedding updates with respect to streaming IoT traffic to avoid entire hypergraph reconstruction and to achieve low-latency IDS for large-scale and evolving IoT ecosystems.

To mitigate the class imbalance problem of IoT traffic and to protect its traffic diversity, more advanced class imbalance techniques will be exploited, including adaptive sampling, cost-sensitive learning, and online imbalance-aware learning, to maintain the traffic diversity and enhance the IDS for large-scale streaming IoT scenarios.

## Data Availability

The original contributions presented in the study are included in the article/supplementary material, further inquiries can be directed to the corresponding author.
